# Medial Axis and Singularities

**DOI:** 10.1007/s12220-017-9763-x

**Published:** 2017-02-28

**Authors:** Lev Birbrair, Maciej P. Denkowski

**Affiliations:** 1 Universidade Federal do Ceará, Fortaleza, Brazil; 2 Faculty of Mathematics and Computer Science, Institute of Mathematics, Jagiellonian University, Łojasiewicza 6, 30-348 Kraków, Poland

**Keywords:** Medial axis, Skeleton, Central set, o-Minimal geometry, Singularities, 32B20, 54F99

## Abstract

This paper is devoted to the study of the *medial axes* of sets definable in polynomially bounded o-minimal structures, i.e. the sets of points with more than one closest point with respect to the Euclidean distance. Our point of view is that of singularity theory. While trying to make the paper self-contained, we gather here also a large bunch of basic results. Our main interest, however, goes to the characterization of those singular points of a definable, closed set $X\subset {\mathbb {R}}^n$, which are reached by the medial axis.

## Introduction

Although the present paper is essentially concerned with sets definable in some o-minimal structure that in addition should be polynomially bounded, many of the results presented herein hold true not only for subanalytic sets (which—recall—do not form an o-minimal structure), but sometimes even in general. This is clearly apparent from the proofs, and we will not stress particularly this fact.

The main motivation for this paper, apart from the fact that medial axes are of utmost importance in pattern recognition and robotics, were the results of [1] and [9]. Despite the fact that the subject of medial axes, central sets, skeletons, cut loci (all these notions denote more or less the same concept and are often incorrectly interchanged) has been extensively studied, astonishingly few results concern the relations to singularities. Our purpose is to study the medial axis on the grounds of singularity theory. The bibliography we present is certainly far from being exhaustive.

## Preliminaries

Throughout this paper, *definable* means *definable in some polynomially bounded o-minimal structure expanding the field of reals* ${\mathbb {R}}$ (for a concise presentation of tame geometry see, e.g. [5]; for simplicity, one can always think about semi-algebraic sets, see also [6]). On the other hand, when we speak of subanalytic sets (see [7] or [8]), then we should keep in mind that they do not form an o-minimal structure unless we control them at infinity (or near the boundary). This does not play any role in the local case, but from the global point of view, the difference is important (see [7] and [9]).

In this section, we present several facts that are of some use. Actually, most of them hold in general, as can be seen from the proofs. The same is true for the results presented in Sect. 2. When the definability is needed, we shall state it clearly.

The main objects we are interested in are the following. Consider a nonempty, closed set $X\subsetneq {\mathbb {R}}^n$ and write

$$\begin{aligned} d(x):=d(x,X)=\inf \left\{ \left| \left| x-y\right| \right| :y\in X\right\} \end{aligned}$$

for the Euclidean distance; it is a globally 1-Lipschitz function. We define the set of closest points to $x\in {\mathbb {R}}^n$ or *supporting set of* *x* as

$$\begin{aligned} m(x):=\left\{ y\in X\mid \left| \left| x-y\right| \right| =d(x,X)\right\} \end{aligned}$$

which is a compact, nonempty subset of *X* intersected with the sphere $\mathbb {S}(x,d(x,X))$. We denote by $\mathbb {B}(x,r)$ the open Euclidean ball centred at *x* and with radius $r>0$ and so $\mathbb {S}(x,r)=\partial \mathbb {B}(x,r)$. For $r=d(x,X)$, we call the sphere or ball *supporting*. Note that *X* cannot enter a supporting ball.

Recall also that any point $x\in \mathbb {B}(a,r)$ where $a\in X$ has its distance *d*(*x*, *X*) realized in $\mathbb {B}(a,2r)$. As already proved, e.g. in [9], *m*(*x*) is a definable multifunction^1^—if *X* is definable.

We write [*x*, *y*] for the segment joining *x* to *y* and $[x,y):=[x,y]\setminus \{y\}$, whenever $x\ne y$.

We shall denote by $C_a(X)$ *the Peano tangent cone* of *X* at $a\in X$, i.e.

$$\begin{aligned} C_a(X)=\left\{ v\in {\mathbb {R}}^n\mid \exists X\ni x_\nu \rightarrow a, t_\nu >0:t_\nu (x_\nu -a)\rightarrow v\right\} , \end{aligned}$$

and write $N_a(X)$ for Clarke’s normal cone of *X* at *a*:

$$\begin{aligned} N_a(X)=\left\{ w\in {\mathbb {R}}^n\mid \forall v\in C_a(X), \langle v, w\rangle \le 0\right\} . \end{aligned}$$

Of course, both sets are definable/subanalytic in the definable or subanalytic case and we have the inequalities $\dim C_a(X)\le \dim _a X$ and $\dim N_a(X)\ge n-\dim _a X$.

We shall be using also some continuity properties, especially of the multifunction *m*(*x*). We briefly recall after [12] the Kuratowski upper and lower limits of a multifunction $F:\mathbb {R}^n\rightarrow \mathscr {P}({\mathbb {R}}^m)$ at a point $x_0$:

- $y\in \liminf _{x\rightarrow x_0} F(x)$ iff for any sequence $x_\nu \rightarrow x_0$, $x_\nu \ne x_0$, one can find a sequence $F(x_\nu )\ni y_\nu \rightarrow y$;
- $y\in \limsup _{x\rightarrow x_0} F(x)$ iff there are sequences $x_\nu \rightarrow x_0$, $x_\nu \ne x_0$, and $F(x_\nu )\ni y_\nu \rightarrow y$.

We take here into account only points from the domain of *F*, i.e. the set of points *x* for which $F(x)\ne \varnothing$ (and $x_0$ is an accumulation point of this domain).

Of course, the upper limit contains the lower one, and both are *closed sets* that do not alter if we replace the values of *F* by their closures. For details in respect of the definable case, see [6]. In particular,

$$\begin{aligned} C_a(X)=\limsup _{\varepsilon \rightarrow 0+} (1/\varepsilon )(X-a). \end{aligned}$$

By the Curve Selection Lemma, in the definable case, we can replace the upper limit by the limit itself (see e.g. [12]).

### Medial Axis, Normal Sets and Central Set

We may see *X* as the boundary of the open set $\Omega ={\mathbb {R}}^n\setminus X$ and thus *d*(*x*, *X*) is the distance to $\partial \Omega$. This is the usual setting for the notions of medial axis and central set used introduced hereafter.

#### Definition 2.1

The *medial axis* of $\Omega$ is the set of points admitting more than one closest point to the boundary:

$$\begin{aligned} M_X:&=\left\{ x\in \Omega \mid \exists y,z\in X:y \ne z, d(x,X)=\left| \left| x-y\right| \right| =\left| \left| x-z\right| \right| \right\} \\&=\left\{ x\in {\mathbb {R}}^n\setminus X\mid \#m(x)>1\right\} . \end{aligned}$$

By the strict convexity of the norm, $M_X$ is nowheredense; besides, it is definable in the definable setting and subanalytic for a subanalytic set *X* (see, e.g. [9]).

There are two more useful sets as far as the realization of the distance is concerned. Namely, *the normal set*

$$\begin{aligned} N(a)=\left\{ x\in {\mathbb {R}}^n\mid a\in m(x)\right\} =\left\{ x\in {\mathbb {R}}^n\mid \left| \left| x-a\right| \right| =d(x,X)\right\} , \> a\in X \end{aligned}$$

and the *univalued normal set*

$$\begin{aligned} N'(a)=\left\{ x\in {\mathbb {R}}^n\mid m(x)=\left\{ a\right\} \right\} , \quad a\in X. \end{aligned}$$

Below we gather some basic and known properties of *N*(*a*) and $N'(a)$:

#### Proposition 2.2

In the introduced setting,

1. $$a\in N'(a)\subset N(a)$$;
2. *N*(*a*) is closed, convex and definable (respectively, subanalytic), actually $X\ni a\mapsto N(a)$ is a definable (resp. subanalytic) multifunction, when *X* is definable (resp. subanalytic);
3. $$N(a)\subset N_a(X)+a$$;
4. $$x\in N'(a)\ \Rightarrow \ [a,x]\subset N'(a)$$ and $$x\in N(a)\setminus \{a\}\ \Rightarrow \ [a,x)\subset N'(a)$$;
5. For any non-isolated $a\in X$, $\limsup _{X\ni b\rightarrow a} N(b)\subset N(a)$;
6. $N'(a)$ is convex and definable/subanalytic (as a set and as a multifunction of $a\in X$) when *X* is definable/subanalytic;

#### Proof

(1) is obvious, (2) is discussed in [15] and [9]. In order to prove that *N*(*a*) is definable or subanalytic as a multifunction, it suffices to observe that its graph coincides with the graph $\Gamma _m$ of *m*(*x*) itself^2^, up to a permutation of coordinates. Next, (3) is to be found in [9], (4) follows from the strict convexity of the norm, and (5) for *N*(*a*) is proved in [9]. To prove (6), we observe that for $x\in [x_1,x_2]$, where $x_1,x_2\in N'(a)$, and we have the inclusion

$$\begin{aligned} \overline{\mathbb {B}}\left( x,\left| \left| x-a\right| \right| \right) \subset \overline{\mathbb {B}}\left( x_1,\left| \left| x_1-a\right| \right| \right) \cup \overline{\mathbb {B}}\left( x_2,\left| \left| x_2-a\right| \right| \right) \end{aligned}$$

and $\overline{\mathbb {B}}(x_j,||x_j-a||)\cap X=\{a\}$. It remains to prove the definability/subanalycity, when *X* is definable/subanalytic. Observe that

$$\begin{aligned}&\left\{ (a,x)\in X\times {\mathbb {R}}^n\mid m(x)=\{a\}\right\} \\&\quad =\left\{ (a,x)\in X\times {\mathbb {R}}^n\mid x\in N(a), \#m(x)=1\right\} \\&\quad =\left\{ (a,x)\mid (x,a)\in \Gamma _m, \dim m(x)=0, \#cc(m(x))=1\right\} \end{aligned}$$

where *cc*(*m*(*x*)) denotes the family of connected components of the set *m*(*x*). Both functions $x\mapsto \dim (\Gamma _m)_x$ and $x\mapsto \#cc((\Gamma _m)_x)$ 3 in the description are definable/subanalytic (for the second one see [6], the set $\Gamma _m$ is *v*-relatively compact) and the assertion follows. $\square$

#### Example 2.3

The set $N'(a)$ need not be closed:

Take $X=\{x^2+y^2=1\}\subset {\mathbb {R}}^2$; then $M_X=\{(0,0)\}$ and for $a=(1,0)$ we have $N'(a)=(0,+\infty )\times \{0\}$ and $N(a)=\overline{N'(a)}$.

#### Example 2.4

It is obvious that $a\in {\mathrm {int}} X$ implies $\#N(a)=1$. We do not have necessarily the converse implication:

Consider $X=\{y\le |x|^{3/2}\}$. Then $M_X=\{0\}\times (0,+\infty )$ (see, e.g. Lemma 3.17) and $N((0,0))=\{(0,0)\}$.

#### Remark 2.5

As is easily seen from the example of $y=|x|$ as *X* and $a=(0,0)$ in (5) from Proposition 2.2, we usually do not have an equality.

Property (5) does not hold for $N'(a)$. First of all, the upper limit is always a closed set, while $N'(a)$ need not be so, as we have just seen. But apart from that, we cannot hope even for an inclusion $\limsup _{X\ni a_\nu \rightarrow a} N'(a_\nu )\subset N'(a)$ as can be seen by taking

$$\begin{aligned} X=\left( (-\infty ,1]\times \{-1,1\}\right) \cup \left\{ (x-1)^2+y^2=1, x\ge 1\right\} \end{aligned}$$

and $a=(1,1)$, $a_\nu =(1-1/\nu ,1)$. Here $N'(a_\nu )=\{a_\nu \}\times (0,+\infty )$ and so the upper limit equals $\{1\}\times [0,+\infty )$, while $N'(a)=\{1\}\times (0,+\infty )$.

Of course, there is always $\limsup _{X\ni b\rightarrow a} N'(b)\subset \overline{N'(a)}$ in view of the next result.

#### Proposition 2.6

We always have $N(a)=\overline{N'(a)}$.

#### Proof

Obviously, $\overline{N'(a)}\subset N(a)$, the latter being closed. Take a point $x\in N(a)\setminus N'(a)$. Then $x\ne a$ and by Proposition 2.2 (4) we have $[a,x)\subset N'(a)$ which ends the proof. $\square$

#### Theorem 2.7

There is

$$\begin{aligned} M_X=\bigcup _{a\in X} N(a)\setminus N'(a)=\bigcup _{a\in X} N(a)\setminus \bigcup _{a\in X} N'(a)={\mathbb {R}}^n\setminus \bigcup _{a\in X} N'(a) \end{aligned}$$

#### Proof

If $x\in M_X$ we take any $a\in m(x)$ and clearly $x\in N(a)\setminus N'(a)$. On the other hand, if $x\in N(a)\setminus N'(a)$, then $a\in m(x)$ is not the only point in *m*(*a*), whence $x\in M_X$.

In the second equality, the set on the left-hand side clearly contains the set on the right-hand side. If $x\in N(a)\setminus N'(a)$, then *m*(*x*) does not reduce to a singleton, and hence $x\notin N'(b)$ for any $b\in X$.

The last equality follows from the obvious observation that there is $\bigcup _{a\in X}N(a)={\mathbb {R}}^n$. $\square$

#### Remark 2.8

Note that $N'(a)\cap N'(b)=\varnothing$ as well as $N'(a)\cap N(b)=\varnothing$, provided $a\ne b$. Under the same condition, we have $N(a)\cap N(b)\subset M_X$, as $M_X=\{x\in {\mathbb {R}}^n\mid \#m(x)\ge 2\}$.

#### Proposition 2.9

In the definable case, the set $X':=\{a\in X\mid N'(a)\subsetneq N(a)\}$ is

1. definable,
2. empty iff $$M_X=\varnothing $$.

#### Proof

Ad(1): Remark that $X'$ is the image of the difference of definable graphs $\Gamma _N\setminus \Gamma _{N'}\subset X\times {\mathbb {R}}^n$ by the projection onto *X*.

Ad (2): This is obvious. $\square$

#### Remark 2.10

Note that $X'$ need not be dense in *X* if only $M_X\ne \varnothing$. Indeed, take $X=(-\infty ,0]\times \{0\}\cup \{(1,0)\}$. Then $X'=\{(0,0), (1,0)\}$.

Another notion closely related to the medial axis is the central set.

#### Definition 2.11

We call $\mathbb {B}(x,r)\subset \Omega$ a *maximal ball* for $\Omega$, if

$$\begin{aligned} \mathbb {B}(x,r)\subset \mathbb {B}(x',r')\subset \Omega \ \Rightarrow \ x=x', r=r'. \end{aligned}$$

The set of the centres of maximal balls for $\Omega$ is called the *central set*. We denote it by $C_X$.

#### Remark 2.12

If $\mathbb {B}(x,r)$ is a maximal ball, then $r=d(x,X)$. Moreover, $C_X$ is clearly definable, if *X* is such (cf. [3]).

There are two natural characterizations of the medial axis and the central set that sometimes prove rather useful:

#### Lemma 2.13

There is

1. $$x\in M_X\ \Leftrightarrow \ \exists a\in m(x):x\notin N'(a)$$;
2. $$x\in C_X\ \Leftrightarrow \ \exists a\in m(x):({\mathbb {R}}_+(x-a)+a)\cap N(a)=[a,x].$$

Moreover, in both cases $\exists$ can be replaced by $\forall$.

#### Proof

The first equivalence is a reformulation of Theorem 2.7 together with the remark following it, as $a\in m(x)$ means exactly $x\in N(a)$. The second one requires a short discussion.

Note that $({\mathbb {R}}_+(x-a)+a)\cap N(a)$ is always a closed segment. If $x\in C_X$, the radius of the maximal ball *B* centred at *x* is *d*(*x*, *X*). Hence, $\overline{B}\cap X=m(x)$. For any point $a\in m(x)$ and any point $y\in ({\mathbb {R}}_+(x-a)+a)\cap N(a)$, we have $[a,y]\subset N(a)$ and so $[a,x]\subsetneq [a,y]$ implies $B\subsetneq \mathbb {B}(y,||y-a||)\subset {\mathbb {R}}^n\setminus X$ contradicting $x\in C_X$.

On the other hand, if $({\mathbb {R}}_+(x-a)+a)\cap N(a)=[a,x]$, then for any ball $\mathbb {B}(x,||x-a||)\subset \mathbb {B}(y,r)\subset {\mathbb {R}}^n\setminus X$ we necessarily have $a\in \partial \mathbb {B}(y,r)$ together with $r=||y-a||$ and so $y\in ({\mathbb {R}}_+(x-a)+a)\cap N(a)$. This implies $y=x$ and so $x\in C_X$. $\square$

One last notion, closely related to the previous ones is that of the *conflict set* of a given finite family of nonempty sets (cf. [1]):

#### Definition 2.14

If $X_1,\dots , X_k\subset {\mathbb {R}}^n$ are closed, pairwise distinct, nonempty sets, where $k\ge 2$, and $\varrho (x):=\min _{i=1}^k d(x,X_i)$, then the set of equidistant points

$$\begin{aligned} {\mathrm {Conf}}(X_1,\dots ,X_k)=\left\{ x\in {\mathbb {R}}^n\mid \exists i\ne j:d(x,X_i)=d(x,X_j)\le \varrho (x)\right\} \end{aligned}$$

is called the *conflict set* of $X_1,\dots , X_k$.

#### Remark 2.15

Usually, the sets $X_i$ in the definition of the conflict set are assumed to be pairwise disjoint. Thanks to this assumption the dimension of the conflict set does not exceed $n-1$ (cf. [1]). Otherwise, we lose some control: consider, e.g. $X_1$, $X_2$ as the half-lines $y=x, x\ge 0$ and $y=-x,x\le 0$, respectively. Then ${\mathrm {Conf}}(X_1,X_2)$ is the union of the half-line $x=0, y\ge 0$ together with the oblique quadrant $y\le -|x|$.

Naturally, the definition makes sense also in any metric space. In particular, if all the sets $X_i$ are contained in $E\subset {\mathbb {R}}^n$, we can compute the relative conflict set ${\mathrm {Conf}}_E(X_1,\dots , X_k)$ with respect to a given metric in *E* (we shall need this later for a sphere with its geodesic metric, cf. Theorem 3.26).

### Some Remarks on the Multifunction *m*(*x*)

We recall the following notion from [12]. Let $F:{\mathbb {R}}^m\rightarrow \mathscr {P}({\mathbb {R}}^n)$ be a multifunction. For $a\in {\mathrm {dom}} F$, we consider

- $F^{-1}(F(a))=\{x\in {\mathbb {R}}^m\mid F(x)=F(a)\}$ *the (strong) pre-image*;
- $F^*(F(a))=\{x\in {\mathrm {dom}} F\mid F(x)\subset F(a)\}$ *the lower pre-image*;
- $F_*(F(a))=\{x\in {\mathbb {R}}^m\mid F(x)\supset F(a)\}$ *the upper pre-image*;
- $F^\#(F(a))=\{x\in {\mathbb {R}}^m\mid F(x)\cap F(a)\ne \varnothing \}$ *the weak pre-image*.

Finally, we may consider a *point pre-image* defined for a point $y\in F(a)$ as the section $(\Gamma _F)_y:=\{x\in {\mathbb {R}}^m\mid y\in F(x)\}$. Obviously,

$$\begin{aligned} F^\#(F(a))=\bigcup _{y\in F(a)}(\Gamma _F)_y. \end{aligned}$$

We are particularly interested in the multifunctions

$$\begin{aligned} m:{\mathbb {R}}^n\ni x\mapsto m(x)\subset X,\quad N:X\ni a\mapsto N(a)\subset {\mathbb {R}}^n \end{aligned}$$

and also $N'$. As already noted, *m* and *N* share the same graph up to a permutation of the coordinates.

We readily observe that for $a\in X$,

1. $$N(a)=(\Gamma _m)_a$$ and $$N'(a)=m^{-1}(m(a))$$ (cf. $$m(a)=\{a\}$$);
2. $N^{-1}(N(a))=\{a\}=m(a)$ (because $N'(a)$ is dense in *N*(*a*) by our previous remarks);
3. $$N'^{*}(N'(a))=N(a)=\overline{N'(a)}$$;
4. $$N^\#(N(a))\supsetneq \{a\}\ \Leftrightarrow \ a\in m(M_X)$$;
5. $$m^\#(m(a))=m^\#(\{a\})=N(a)$$, while for $$b\notin X$$, $$m^{\#}(m(b))=\bigcup _{y\in m(b)} N(y)$$.

Moreover, for $$a\in {\mathbb {R}}^n$$,

1. $$a\in M_X\Rightarrow m_*(m(a))\subset M_X$$;
2. $$a\notin M_X\Rightarrow m^*(m(a))\cap M_X=\varnothing $$.

Recall that for a multifunction *F* as above and $G:{\mathbb {R}}^n\rightarrow \mathscr {P}({\mathbb {R}}^p)$ we define the composition $G\circ F$ by

$$\begin{aligned} (x,z)\in \Gamma _{G\circ F}\ \Leftrightarrow \ \exists y\in {\mathbb {R}}^n:(x,y)\in \Gamma _F\ {\text {and}}\ (y,z)\in \Gamma _G. \end{aligned}$$

Let us compose *m* with *N* in two ways. First for $(x,z)\in {\mathbb {R}}^n\times {\mathbb {R}}^n$

$$\begin{aligned}&(x,z)\in \Gamma _{N\circ m} \Leftrightarrow \ \exists y\in X:y\in m(x)\ {\text {and}}\ z\in N(y)\\&\quad \Leftrightarrow \ \exists y\in X:y\in m(x)\ {\text {and}}\ y\in m(z)\\&\quad \Leftrightarrow \ m(x)\cap m(z)\ne \varnothing \\&\quad \Leftrightarrow \ z\in m^\#(m(x)). \end{aligned}$$

In particular, if $x,z\in N(a)$, then $(x,z)\in \Gamma _{N\circ m}$.

Next, for $$(a,b)\in X\times X$$,

$$\begin{aligned}&(a,b)\in \Gamma _{m\circ N} \Leftrightarrow \ \exists x\in {\mathbb {R}}^n:x\in N(a)\ {\text {and}}\ b\in m(x)\\&\quad \Leftrightarrow \ \exists x\in {\mathbb {R}}^n:a,b\in m(x). \end{aligned}$$

In particular, if we denote $\Delta :=\{(x,x)\mid x\in X\}$ the diagonal in $X\times X$, we obtain $\Delta \subset \Gamma _{m\circ N}$ and

$$\begin{aligned} M_X=\varnothing \ \Leftrightarrow \Gamma _{m\circ N}=\Delta \ \Leftrightarrow \ m\circ N={\mathrm {id}}_X. \end{aligned}$$

By the results of [12] Section 6, we have a Łojasiewicz-type inequalities in the definable setting:

#### Proposition 2.16

Let *F* denote either the closed multifunction *N*(*x*), $x\in X$, or the compact one *m*(*x*), $x\in {\mathbb {R}}^n$. Then for any point $x_0$ in the domain of *F*, there are constants $C,\ell >0$ such that in a neighbourhood of $x_0$,

$$\begin{aligned} {{\mathrm {dist}}}_{HK}(F(x),F(x_0))\ge C d(x,F^\bullet (F(x_0)))^\ell \end{aligned}$$

where ${\mathrm {dist}}_{HK}$ denotes the Hausdorff-Kuratowski distance^4^, and $F^\bullet (F(x_0))$ stands for any of the pre-images introduced above. In particular,

1. ${\mathrm {dist}}_K(N(x),N(x_0))\ge C ||x-x_0||^\ell$ for all $x\in X$ near $x_0\in X$;
2. ${\mathrm {dist}}_H(m(x),m(x_0))\ge C d(x,N'(x_0))^\ell$ for all $x\in {\mathbb {R}}^n$ near $x_0\in X$;
3. ${\mathrm {dist}}_H(m(x),m(x_0))\ge C d(x,N(x_0))^\ell$ for all $x\in {\mathbb {R}}^n$ near $x_0\in X$;
4. ${\mathrm {dist}}_H(m(x),m(x_0))\ge C d(x,\bigcup _{y\in m(x_0)} N(y))^\ell$ for all $x\in {\mathbb {R}}^n$ near $x_0\in {\mathbb {R}}^n$;
5. ${\mathrm {dist}}_H(m(x),m(x_0))\ge C d(x,M_X)^\ell$ for all $x\in {\mathbb {R}}^n$ near $x_0\in M_X$

#### Proof

The result follows directly from [12] Corollary 6.4. To prove the particular cases, we use the computations made above for the strong pre-image of *N* of $x_0\in X$ (this gives (1)), the strong pre-image of *m* of $x_0\in X$ and the fact that $\overline{N'(x_0)}=N(x_0)$ (this gives (2) and (3)); the weak pre-image of *m* gives (4); (5) follows from $m_*(m(x_0))\subset M_X$ when $x_0\in M_X$. $\square$

Now, we can add a property of *m*(*x*) that is useful in this context (no definability is needed).

#### Proposition 2.17

The multifunction *m*(*x*) is upper semi-continuous: $\limsup _{D\ni x\rightarrow x_0} m(x)=m(x_0)$ at any point $x_0\in {\mathbb {R}}^n$ and for any dense subset *D* of ${\mathbb {R}}^n$.

#### Proof

If $y=\lim y_\nu$ for some $y_\nu \in m(x_\nu )$ where $D\ni x_\nu \rightarrow x_0$, then $||y_\nu -x_\nu ||=d(x_\nu ,X)$ and so passing to the limit yields $y\in m(x_0)$.

As for the converse inclusion, by [12], we have nothing to prove, if $m(x_0)$ reduces to a point. Now, if $y\in m(x_0)$, we can assume that $[x_0,y]\setminus \{x_0\}$ is nonempty. Take neighbourhoods $U\ni y$ and $V\ni x_0$. Then $\varnothing \ne V\cap [x_0,y]\setminus \{x_0\}\subset N'(y)$ (cf. Proposition 2.2 (4)). Hence, arbitrarily near $x_0$, we can find points *x* with $m(x)=\{y\}$. But *D* is dense, so arbitrarily near *x* there are points $x'\in D$ and as $\#m(x)=1$, again by [12] we have the continuity of *m* at *x*. Thus, there is a point $x'\in D\cap V$ for which $m(x')\cap U\ne \varnothing$. We have shown that $\limsup _{D\ni x\rightarrow x_0} m(x)=m(x_0)$. $\square$

#### Remark 2.18

Note that along $M_X$ we usually only have an inclusion (see [9]): $\limsup _{M_X\ni x\rightarrow x_0} m(x)\subset m(x_0).$

Another useful fact is the following observation that holds in general.

#### Proposition 2.19

Let $U\subset {\mathbb {R}}^n$ be open and nonempty. Assume that there is a continuous selection $\mu :U\rightarrow X$ for *m*(*x*), i.e. for any $x\in U$, $\mu (x)\in m(x)$. Then $\mu =m|_U$, i.e. *m*(*x*) is univalent on *U*.

#### Proof

Obviously, by the preceding Proposition, $\mu$ coincides with *m* on $U\setminus M_X$. Suppose that there is a point $x\in U\cap M_X$. Then by Theorem 2.7, $x\in N(a)\setminus N'(a)$ for some $a\in X$. From the definition of the normal set, it follows that $x\in N(b)$ for some $b\in X\setminus \{a\}$. Then when we approach *x* along the two distinct segments [*a*, *x*] and [*b*, *x*], we get two different limits for $\mu$, contrary to the continuity. $\square$

### On the Distance Function and the Medial Axis

As already observed in [17] (without a proof), the set $M_X$ is related in a most natural way (based on the results of [4]) to the singularities (non-differentiability points) of the function $\delta (x):=d(x,X)^2$. Namely, we give a simple proof of the Theorem 2.23 below. Before we do it, we recall that for any locally Lipschitz function $f:U\rightarrow {\mathbb {R}}$ with $U\subset {\mathbb {R}}^n$ open, the Rademacher Theorem asserts that the set $D_f$ of differentiability points is dense in *U*. Hence, we can define the *Clarke subdifferential* $\partial f(x)$ at any point $x\in U$ as the convex hull ${\mathrm {cvx}} \nabla _f(x)$ of the set $\nabla _f(x)$ of all the possible limits of the gradients $\nabla f(x_\nu )$ for sequences $D_f\ni x_\nu \rightarrow x$. It is easy to see that $\partial f(x)$ is a compact set, and by [4], it reduces to a point *y* iff $x\in D_f$ and $\nabla f|_{D_f}$ is continuous at *y* (then $\partial f(x)=\{y\}$). Of course, to compute $\partial f(x)$, we may restrict ourselves to any dense subset of $D_f$ (see [4]). Some discussion of $x\mapsto \partial f(x)$ as a multifunction is presented in [12]. For the moment, we do not need any definability assumptions.

#### Lemma 2.20

([4]) If $d(x)=d(x,X)$ is differentiable and non-zero at a point $x_0$, then $x_0\notin X$, $\#m(x_0)=1$ and $\nabla d(x_0)=\frac{x_0-m(x_0)}{d(x_0)}$.

Actually, we will need a slightly altered version of this lemma:

#### Lemma 2.21

The function *d*(*x*) is differentiable at any $x_0\notin {M_X}\cup X$ and $\nabla d(x_0)=\frac{x_0-m(x_0)}{d(x_0)}$.

#### Proof

Since $U={\mathbb {R}}^n \setminus (\overline{M_X}\cup X)$ is open, the set of differentiability points of *d* in *U*, $D_d\cap U$, is dense in *U*. But $U\subset {\mathbb {R}}^n\setminus (M_X\cup X)\subset {\mathbb {R}}^n\setminus X$ and we know that $M_X$ is nowheredense in ${\mathbb {R}}^n$ and $D_d\cap {\mathbb {R}}^n\setminus X$ is dense in ${\mathbb {R}}^n\setminus X$. Hence $D_d\cap {\mathbb {R}}^n\setminus (M_X\cup X)$ is dense in $U'={\mathbb {R}}^n\setminus (M_X\cup X)$. The mapping $v(x)=\frac{x-m(x)}{d(x)}$ is well-defined and continuous in $U'$. Moreover, $d(x+tv(x))=||x-m(x)||+t$ for $x\in U'$ and small $t<0$ (then $x+tv(x)\in U'$). Therefore, for any $x\in D_d\cap U'$,

$$\begin{aligned} \langle \nabla d(x),v(x)\rangle =\lim _{t\rightarrow 0-}\frac{d(x+tv(x))-d(x)}{t}=1, \end{aligned}$$

while the left-hand side is smaller than $||\nabla d(x)||$ by the Cauchy-Schwarz inequality. Observe that *d*(*x*) being 1-Lipschitz we have $||\nabla d(x)||\le 1$ at any differentiability point $x\in D_d$ 5, so $||\nabla d(x)||=1$. Note also that $\langle u,w\rangle =||u||\cdot ||w||\cos \angle (u,w)$ and so in view of the fact that we get $\langle \nabla d(x),v(x)\rangle =||\nabla d(x)||=1$, we conclude that $\nabla d(x)=\lambda v(x)$ for some $\lambda >0$ 6. Again $||v(x)||=1$ implies $\lambda =1$ and we are done. By the continuity of *v*(*x*), the formula holds in the whole of $U'$ (the only possible value for the subdifferential $\partial d(x)$ at $x\in U'$ is *v*(*x*) and $\nabla d(x)$ varies continuously on $D_d\cap U'$ due to the continuity of *v*(*x*)). Clarke’s results ([4, Proposition 1.13]) give the assertion. $\square$

Of course, the function $\delta (x)=d(x)^2$ is a locally Lipschitz function. This function encodes information about the singularities of *X*, see [15]. Moreover, we can compute $\nabla \delta (x)$ also for points $x\in X$, getting zero. This leads to:

#### Lemma 2.22

The function $\delta (x)$ is differentiable at any point $x_0\notin M_X$ and $\nabla \delta (x_0)=2(x-m(x_0))$. Moreover, $\delta (x)$ is of class ${\mathcal {C}}^1$ in the open set ${\mathbb {R}}^n\setminus \overline{M_X}$.

#### Proof

The set of differentiability points $D_\delta$ contains the analogous set $D_d$ of *d*. By the previous Lemma, for any $x\notin M_X\cup X$, we can compute

$$\begin{aligned} \nabla \delta (x)=2d(x)\frac{x-m(x)}{d(x)}=2(x-m(x)). \end{aligned}$$

On the other hand, for any point $x_0\in X$, since $d(x)=|d(x)-d(x_0)|\le ||x-x_0||$, we get $\delta (x)=|\delta (x)-\delta (x_0)|< \varepsilon ||x-x_0||$, provided $||x-x_0||<\varepsilon$. This implies that $\nabla \delta (x_0)=0=2(x_0-m(x_0))$, too.

The last part of the Lemma follows from [4] together with Proposition 2.17. $\square$

Part of the following theorem appeared as Proposition 2.2 in [17] without a proof. In order to make our paper self-contained, we give a direct proof—independent of [4] Theorem 2.1 evoked in [17]—of a slightly extended version of this result^7^.

#### Theorem 2.23

We have for any point $x\in {\mathbb {R}}^n$,

1. $$\partial \delta (x)=\{2(x-y)\mid y\in {\mathrm {cvx}}\ m(x)\}$$;
2. The following conditions are equivalent:
  - $$x\in M_X$$;
  - $$\#\partial \delta (x)>1$$;
  - $$x\notin D_\delta $$;
  - $$x\notin D_d\cup X$$.
3. $$\nabla \delta (x)=0\Leftrightarrow x\in X$$;
4. $\nabla \delta$ is continuous in $D_\delta ={\mathbb {R}}^n\setminus M_X$.

#### Proof

Clearly, in order to get the equivalence (a)$\Leftrightarrow$(b), we need only to show the formula for $\partial \delta (x)$. If $x\notin {M_X}$, then the last Lemma gives $\nabla \delta (x)=2(x-m(x))$. Moreover, we know that $D={\mathbb {R}}^n\setminus M_X$ is dense in ${\mathbb {R}}^n$. Clearly, a sequence $\{\nabla \delta (x_\nu )\}$ for $\{x_\nu \}\subset D$ converging to some $x_0$, has a limit if and only $\{m(x_\nu )\}$ has a limit. Therefore,

$$\begin{aligned} \partial \delta (x_0)&={\mathrm {cvx}}\left\{ 2(x-y)\mid y\in \limsup _{D\ni x\rightarrow x_0} m(x)\right\} \\&=\left\{ 2(x-y)\mid y\in {\mathrm {cvx}} \limsup _{D\ni x\rightarrow x_0} m(x)\right\} . \end{aligned}$$

Proposition 2.17 ends the proof of (1).

From the previous Lemmas, we know that (c)$\Rightarrow$(a), (d)$\Rightarrow$(a) (Lemma 2.21 and the fact that $M_X\cap X=\varnothing$) and that $D_d\cup X\subset D_\delta$, i.e. (c)$\Rightarrow$(d). Now, we will show that $x\in D_\delta$ implies $x\notin M_X$. Taking $x\in D_\delta$ we may assume that $x\notin X$ and so $x\notin m(x)$. Let $y\in m(x)$ and denote by $x_t=y+t(x-y)=x+(1-t)(y-x)$ a point with $t\in (0,1)$. We have $\delta (x_t)=||x_t-y||^2=t^2||x-y||^2$ and we may write for *t* close to 1,

$$\begin{aligned} \delta (x_t)-\delta (x)&=\langle \nabla \delta (x),x_t-x\rangle +o(\left| \left| x_t-x\right| \right| )\\ \Rightarrow \delta (x)(t^2-1)&=\langle \nabla \delta (x),(1-t)(y-x)\rangle +o\left( (1-t)\left| \left| y-x\right| \right| \right) \\ \Rightarrow -\delta (x)(t+1)&=\langle \nabla \delta (x),y-x\rangle +\frac{o(1-t)}{1-t}. \end{aligned}$$

Making *t* tend to 1, we obtain $-2\delta (x)=\langle \nabla \delta (x),y-x\rangle$. The left-hand side is non-zero which implies $\nabla \delta (x)\ne 0$ and proves (3) (cf. the last Lemma). Then, $d=\sqrt{\delta }$ is differentiable at *x* with $\nabla d(x)=\frac{\nabla \delta (x)}{2d(x)}$. This yields

$$\begin{aligned} 2\left| \left| x-y\right| \right|&=2d(x)\left| \left| \nabla d(x)\right| \right| \cos \angle (\nabla \delta (x),x-y)\Rightarrow \\ 1&=\left| \left| \nabla d(x)\right| \right| \cos \angle (\nabla \delta (x),x-y). \end{aligned}$$

But $||\nabla d(x)||\le 1$ and so we obtain $||\nabla d(x)||=1$ and $\cos \angle (\nabla \delta (x),x-y)=1$. Therefore, $\nabla \delta (x)=2(x-y)$ which implies that $m(x)=\{y\}$ as required. By the way, this shows the inclusion $D_\delta \setminus X\subset D_d$.

Finally, (4) follows from the previous Lemma combined with Proposition 2.17, the equivalence (a)$\Leftrightarrow$(c) (or simply by [4] and (b)$\Leftrightarrow$(c)). $\square$

#### Remark 2.24

At $x\in D_d\subset D_\delta$, we have $\nabla \delta (x)=2d(x)\nabla d(x)$, and from (3), we see that the discontinuity points of $\nabla d$ must belong to $\partial X$. To be more precise, $\partial {\mathrm {int}} X\subset \partial X$ are certainly discontinuity points of $\nabla d$ since we can reach them both from the interior of *X* (where the gradient vanishes) and from ${\mathbb {R}}^n\setminus X$ (where the gradient has norm 1). On the other hand, isolated points of *X* do not belong to $D_d$.

Note that on $D_\delta ={\mathbb {R}}^n\setminus M_X$ the multifunction *m*(*x*) is univalued and we have the obvious relation $\delta (x)=||x-m(x)||^2$. However, *m* need not be differentiable there as we can easily see by taking $X=\{xy=0, x,y\ge 0\}\subset {\mathbb {R}}^2$ and the point $(0,-1)\in \partial N(0,0)$.

Finally, remark that whenever *m*(*x*) is univalued, we have the formula

$$\begin{aligned} m(x)=x-\frac{1}{2}\nabla \delta (x) \end{aligned}$$

which apart from *X* can be rewritten also as

$$\begin{aligned} m(x)=x-d(x)\nabla d(x). \end{aligned}$$

In particular, we conclude that basic differential properties of *m* are related to the Hessian of $\delta$.

The relation between the medial axis and the central set is known (see [13] and the citation in [3]) and given in the following theorem which we prove in a different way than it is done in [13]. All the same, our proof is most probably a standard one.

#### Theorem 2.25

For a closed, nonempty set $X\subsetneq {\mathbb {R}}^n$,

$$\begin{aligned} M_X\subset C_X\subset \overline{M_X}. \end{aligned}$$

#### Proof

The first inclusion is obvious: if $x\in M_X$, then the set $m(x)\subset \mathbb {S}(x,d(x,X))$ consists of more than one point, and the boundary of any ball $\mathbb {B}(x,d(x,X))\subset \mathbb {B}(y,r)\subset {\mathbb {R}}^n\setminus X$ has to be tangent to $\mathbb {S}(x,d(x,X))$ at any point of *m*(*x*). Hence $\mathbb {B}(x,d(x,X))=\mathbb {B}(y,r)$.

For the second inclusion, we have more to do. Consider the open set $U:={\mathbb {R}}^n\setminus (\overline{M_X}\cup X)$. Then $x\mapsto m(x)$ is univalued, and by Lemma 2.21 the gradient $\nabla d(x)=(x-m(x))/d(x)\in \mathbb {S}^1$ is well-defined and continuous in *U* (cf. Proposition 2.17).

Fix $a\in U$ and observe that for any $x\in (m(a),a]\subset U$ we have $\nabla d(x)=\nabla d(a)$. Let us parametrize the segment by arc-length, i.e. $x(t):=m(a)+(t/d(a))(a-m(a))$ for $t\in (0,d(a)]$. We obtain

$$\begin{aligned} x'(t)=\nabla d(a)=\nabla d(x(t)),\quad t\in (0,d(a)). \end{aligned}$$

In other words, *x*(*t*) is the solution of the equation $x'=\nabla d(x)$ (in *U*) with initial condition $x(d(a))=a$. The right-hand side is continuous, whence the Cauchy-Peano Theorem guarantees that *x*(*t*) can be extended as $\gamma (t)$ to some interval $(0,d(a)+\varepsilon )$. Put $\gamma (0)=m(a)$ extending $\gamma$ continuously to $[0,d(a)+\varepsilon )$.

Since $||\nabla d(x)||=1$, this extended integral curve $\gamma (t)$ is automatically parametrized by arc-length and so has length $d(a)+\varepsilon$. If we prove that 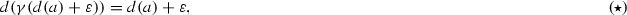


then in view of the fact that $\gamma (0)=m(a)\in X$ we can conclude that $\gamma$ coincides with the segment $[m(a),\gamma (d(a)+\varepsilon ))$. This implies that $\gamma (d(a)+\varepsilon )$ is in *N*(*m*(*a*)) (even in $N'(m(a))$ due to the definition of the set *U* where $\gamma$ lives) and so $a\notin C_X$ 8.

Actually, $(\star )$ follows from an easy computation:

$$\begin{aligned} d\left( \gamma (d(a)+\varepsilon )\right)&=\int _0^{d(a)+\varepsilon } \frac{d}{dt}d(\gamma (t))\ dt\\&=\int _0^{d(a)+\varepsilon }\langle \nabla d(\gamma (t)),\gamma '(t)\rangle \ dt\\&=\int _0^{d(a)+\varepsilon }\left| \left| \nabla d(\gamma (t))\right| \right| ^2\ dt\\&=d(a)+\varepsilon . \end{aligned}$$

The discussion above shows that $C_X\subset \overline{M_X}\cup X$. However, by definition, $C_X\cap X=\varnothing$ and so we are done. $\square$

#### Remark 2.26

The inclusions may be strict: for the first one consider $X=\{y=x^2\}$ in ${\mathbb {R}}^2$ (then the focal point $(0,1/2)\in C_X\setminus M_X$), for the second one see [3] Example 2.2.

These inclusions, although simple, prove often useful, for example:

#### Theorem 2.27

Let $\Omega \subset {\mathbb {R}}^n$ be open and such that it does not contain a half-space^9^, $X:=\partial \Omega$. Then $M_X\ne \varnothing$.

#### Proof

Pick a point $x\in \Omega$. If $x\notin M_X$ then there is exactly one point $y\in X$ realizing $d:=d(x,X)$. For $t>0$, let $x_t=y+t(x-y)$ and we put

$$\begin{aligned} r:=\sup \left\{ t>0\mid \mathbb {B}(x_t,td)\subset \Omega \right\} . \end{aligned}$$

Then $r\ge 1$ is finite since $\Omega$ does not contain a half-space. We will show that $x_r\in \overline{M_X}$. Note that we necessarily have $r=d(x_r,X)$.

Suppose that $x_r\notin \overline{M_X}$. Then, since $C_X\subset \overline{M_X}$, we would find a ball $\mathbb {B}(z,\rho )\subset \Omega$ containing $\mathbb {B}(x_r,r)$. But as $y\in \overline{\mathbb {B}}(x_r,r)\subset \overline{\mathbb {B}}(z,\rho )$, we must have $y\in \partial \mathbb {B}(z,\rho )$. But this implies that $z=x_t$ for some $t>r$ which contradicts the definition of *r*. $\square$

#### Remark 2.28

Taking the closure in the proof is necessary as we can see from the example $X=\{v=u^2\}$ with $y=(0,0)$; we obtain $r=1/2$ and $x_r=(0,1/2)\in \overline{M_X}\setminus M_X$.

## Reaching of Singularities of Hypersurfaces

Let us fix some more notation: for $k\in \mathbb {N}\cup \{\infty ,\omega \}$ we put

$$\begin{aligned} {\mathrm {Reg}}_k X:=\left\{ x\in X\mid X\> \hbox {is a}\> {\mathcal {C}}^k-\hbox {submanifold at}\> x\right\} \end{aligned}$$

and ${{\mathrm {Sng}}}_k X:=X\setminus {\mathrm {Reg}}_k X$. In the analytic case, i.e. for $k=\omega$, we omit the index.

Of course, even a plane analytic curve can have almost any type of singularity in the sense that $0\in {{\mathrm {Sng}}} X$ if and only if either $\Gamma$ has a cusp at zero, or there is an integer $k\ge 1$ such that $0\in {\mathrm {Reg}}_k X\cap {{\mathrm {Sng}}}_{k+1} X$ and all the possibilities can occur:

### Example 3.1

Take two relatively prime integers $p>q$ such that for a given *k* we have $k<p/q<k+1$ and consider the curve *X* defined by $y^q=x^p$. Then $0\in {\mathrm {Reg}}_k X\cap {{\mathrm {Sng}}}_{k+1} X$. For instance the function $y=x^{5/3}$ has analytic graph and is ${\mathcal {C}}^1$ but not ${\mathcal {C}}^2$ smooth at the origin.

The starting point of our considerations is the observation made in [9] that there are natural instances when $M_X$ reaches the singularities of *X*. However, this does not happen always.

### Example 3.2

Consider in ${\mathbb {R}}^2$ the sets $\Omega _1:=\{y>x^2\}$, $\Omega _2:=\{y>|x|^{3/2}\}$ and $\Omega _3:=\{y>(1+{\mathrm {sgn}} x)x^2\}$. Then the boundaries $X_i$, $i=2,3$ are ${\mathcal {C}}^2$ smooth except at the origin where they are only ${\mathcal {C}}^1$ smooth, while $X_1$ is ${\mathcal {C}}^2$-smooth everywhere.

It is easy to see that $M_{X_1}$ is the half-line $\{0\}\times (1/2,+\infty )$ and so it does not meet $X_1$. On the other hand, $M_{X_2}=\{0\}\times (0,+\infty )$ reaches the ${\mathcal {C}}^1$-singularity of $X_2$. But again $M_{X_3}$ stays away from it. This is due to the fact that although both $X_2$ and $X_3$ have the same kind of singularity, their geometric radii of curvature (we will define this precisely later on) are different.

### Remark 3.3

It is worth to note at this point that for a given $a\in X$, we have

$$\begin{aligned} M_X\cap \mathbb {B}(a,r)=M_{X\cap \mathbb {B}(a,2r)}\cap \mathbb {B}(a,r), \end{aligned}$$

since any point $x\in \mathbb {B}(a,r)$ has its distance to *X* realized in $X\cap \mathbb {B}(a,2r)$.

We will need the following result completing a Lemma of Yomdin (Part 3, Lemma 1 in [17]):

### Lemma 3.4

Let $X\subset {\mathbb {R}}^n$ be a closed, nonempty set, $x_0\in {\mathbb {R}}^n\setminus X$ a point and $B=\mathbb {B}(x_0,r)$ a ball such that $\overline{B}\cap X=\bigcup _{j=1}^k X_j$ where the sets $X_j$ are nonempty and pairwise disjoint, and for at least one *i*, $X_i\cap B\ne \varnothing$. Then there exists a neighbourhood *U* of $x_0$ such that

$$\begin{aligned} d(x,X)=\min _{j=1}^k d(x,X_j), \quad x\in U. \end{aligned}$$

### Proof

Write $d(x)=d(x,X)$ and $d_j(x)=d(x,X_j)$. Clearly, $d(x)\le d_j(x)$ which gives one inequality. Note that $d(x_0)=\min _{j=1}^k d_j(x_0)$ 10.

Suppose that there is a sequence $x_\nu \rightarrow x_0$ such that

$$\begin{aligned} d(x_\nu )<\min _{j=1}^kd_j(x_\nu ). \end{aligned}$$

By passing to a subsequence, we may assume that $\min _{j=1}^kd_j(x_\nu )=d_1(x_\nu )$ for all $\nu$. We can find points $y_\nu \in X\setminus \overline{B}$ and $z_\nu \in X_1$ such that $||x_\nu -y_\nu ||=d(x_\nu )$ and $||x_\nu -z_\nu ||=d_1(x_\nu )$. Since $X_1$ is compact, by extracting a subsequence, we may assume that $z_\nu \rightarrow z_0\in X_1$. Similarly, in view of the inequality $||y_\nu ||\le ||y_\nu -x_\nu ||+||x_\nu ||$, we see that the sequence $\{y_\nu \}$ is bounded and again we may assume that $y_\nu \rightarrow y_0$. Clearly $y_0\in X\setminus B$.

Now, observe that $||x_0-y_0||=||x_0-z_0||$ which implies that $y_0, z_0\in \partial B$ and hence $d(x_0)=r$. But this means that for any *j*, $X_j\subset \partial B$ contrary to the assumptions. $\square$

### Remark 3.5

In the situation of the Lemma above, we have in particular that $M_X\cap U={\mathrm {Conf}}(X_1,\dots , X_k)\cap U$.

### General Remarks on Hypersurface Singularities

We suppose that *X* is locally homeomorphic to ${\mathbb {R}}^{n-1}$.

#### Lemma 3.6

Assume that $x_1,x_2\in {\mathbb {R}}^n\setminus M_X$ are different but $m(x_1)=m(x_2)=a$. If, moreover, the vectors $x_1-a$ and $x_2-a$ are linearly independent, then $a\in {{\mathrm {Sng}}}_1 X$.

#### Proof

The two hyperplanes $H_i$ defined by the vectors $x_i-a$ are different. If we had $a\in {\mathrm {Reg}}_1 X$, then by the choice of *a*, we would have $(x_i-a)\bot T_a X$ for $i=1,2$, whence $T_a X=H_1=H_2$ which is a contradiction. $\square$

#### Proposition 3.7

Assume that in a neighbourhood *U* of $x_0\in {\mathbb {R}}^n$ we have $m\equiv a\in X$. Then $a\in {{\mathrm {Sng}}}_1 X$.

#### Proof

We know that $M_X$ is nowheredense, hence $U\setminus M_X\ne \varnothing$. Thus, we can apply the preceding Lemma to some points $x_1, x_2$. $\square$

#### Proposition 3.8

If $x_0\in \mathbb {R}^n\setminus \overline{M_X}$ is such that $m(x_0)\in {\mathrm {Reg}}_1 X$, then for $S=B\cap \mathbb {S}(x_0,d(x_0))$ where *B* is a sufficiently small open ball centred at $x_0$, the mapping $m|_S:S\rightarrow m(S)$ is a homeomorphism onto the open subset $m(S)\subset {\mathrm {Reg}}_1 X$.

#### Proof

By assumption, *m* is univalued in a neighbourhood *U* of $x_0$ and so also continuous. Thus, if we fix a neighbourhood $V\ni m(x_0)$ such that $V\cap X$ is ${\mathcal {C}}^1$-smooth, we can find a ball ${B}\subset U$ centred at $x_0$ such that $m({B})\subset V$. By Proposition 3.7, for two points $x_1,x_2\in \overline{S}$ with $m(x_1)=m(x_2)=a$, we necessarily have an inclusion between the segments $[x_1,a]$, $[x_2,a]$ which implies (provided *B* is small enough) that $x_1=x_2$. Thus, $m|_S$ is injective, and so by the Brouwer Domain Invariance Theorem, $m|_S$ is a homeomorphism onto the open set $m(S)\subset {\mathrm {Reg}}_1 X$. $\square$

Recall that in case $X={\mathrm {Reg}}_2 X$, for any point $a\in X$ and $x_0$ belonging to the normal line $(T_aX)^\bot +a$ to *X* at *a*, the function $\phi (y)=||x_0-y||^2$, $y\in X$, has a local minimum at $y=a$ if and only if there is no focal point on $[a,x_0]\setminus \{x_0\}$ (see [16]^11^).

Observe that having a local minimum at *a* does not necessarily mean that *a* realizes the distance of $x_0$ to *X*. However, if *a* is a local minimum in $\mathbb {B}(a,r)\cap X$ and *r* satisfies $||a-x_0||<r/2$, then indeed $\phi (a)=d(x_0,X)$.

### Superquadratic Functions

Motivated by the examples considered so far, we introduce the notion of *superquadraticity*.

Let *X* be the graph of a non-negative continuous function germ $f:({\mathbb {R}}^n,0)\rightarrow ({\mathbb {R}}_+,0)$.

#### Definition 3.9

In this situation, we call *X* *superquadratic* at the origin, if the function $g_f(r):=\max _{x\in \mathbb {S}(0,r)} f(x)$ is superquadratic, i.e. it can be written near zero as $g(r)=ar^\alpha +o(r^\alpha )$ with $0<\alpha <2$ (in particular *f* is non-constant).

There are two other closely related definitions:

#### Definition 3.10

We define the *order at zero* of a continuous definable function germ $f:({\mathbb {R}}^n,0)\rightarrow ({\mathbb {R}},0)$ as

$$\begin{aligned} {\mathrm {ord}}_0 f=\sup \left\{ \eta >0\mid \left| f(x)\right| \le {{\mathrm {const.}}} \left| \left| x\right| \right| ^\eta , \left| \left| x\right| \right| \ll 1\right\} , \end{aligned}$$

if $f\not \equiv 0$, and ${\mathrm {ord}}_0 f:=+\infty$ otherwise.

#### Remark 3.11

Clearly, the definition is well-posed since we are in a polynomially bounded o-minimal structure, by the Łojasiewicz inequality. It is a mere exercise to prove that in one variable $g(t)=at^\alpha +o(t^\alpha )$ is written precisely with $\alpha ={\mathrm {ord}}_0 g$ and $|g(t)|\le {{\mathrm {const.}}} |t|^\alpha$.

By the methods used by Bochnak and Risler in [2] Theorem 1, it is easy to show that the least upper bound in the definition is in fact attained.

Note also that the inequality defining the order is satisfied with any exponent $\alpha \le {\mathrm {ord}}_0 f$ and that it makes sense also for a vector-valued *f*; then it is written as $||f(x)||\le {{\mathrm {const.}}} ||x||^\eta$. In the latter case, ${\mathrm {ord}}_0 f$ coincides with the minimal order of the components $f_i$ of $f=(f_1,\dots , f_k)$ 12.

#### Definition 3.12

We call *sectional order at zero* for a definable function $f:({\mathbb {R}}^n,0)\rightarrow ({\mathbb {R}},0)$, $f\not \equiv 0$, the number

$$\begin{aligned} s_0(f)=\inf \left\{ \alpha >0\mid f(tv)=at^\alpha +o(t^\alpha ), 0\le t\ll 1, v\in \mathbb {S}^{n-1}:f\mid _{{\mathbb {R}}_+v}\not \equiv 0\right\} . \end{aligned}$$

#### Proposition 3.13

Consider a non-constant, continuous, definable germ $f:({\mathbb {R}}^{n},0)\rightarrow ({\mathbb {R}},0)$. Then for the following three conditions:

1. $$s_0(f)<2$$;
2. $${\mathrm {ord}}_0 f<2$$;
3. |*f*| is superquadratic at 0;

we have $$(1)\Rightarrow (2)\Leftrightarrow (3)$$.

#### Proof

The implications $(1)\Rightarrow (2)\Rightarrow (3)$ are immediate. Indeed, if (2) does not hold, then in a neighbourhood of zero, $|f(x)|\le C||x||^2$ for some $C>0$. Thus, for *f*(*tv*) we have for all $t\ge 0$ small enough, $|at^\alpha +o(t^\alpha )|\le Ct^2$ which implies $\alpha \ge 2$ (divide both sides by $t^\alpha$ and take $t\rightarrow 0^+$) and so $s_0(f)\ge 2$. If (3) does not hold, then $|f(x)|\le g_{|f|}(||x||)=a||x||^\alpha +o(||x||^\alpha )$ for some $\alpha \ge 2$. But as ${\mathrm {ord}}_0 g_{|f|}=\alpha$, we obtain $|f(x)|\le {{\mathrm {const.}}}||x||^\alpha$ and so ${\mathrm {ord}}_0 f\ge 2$.

Furthermore, to see that $(3)\Rightarrow (2)$ suppose that ${\mathrm {ord}}_0 f\ge 2$ and consider the definable set $A=\{(r,x)\in [0,\varepsilon ]\times {\mathbb {R}}^{n}\mid ||x||=r, g_{|f|}(||x||)=|f(x)|\}$. Then 0 is an accumulation point of *A* and so there is a continuous definable selection $r\mapsto (r,\gamma (r))\in A$.

Then $g_{|f|}(r)=|f(\gamma (r))|$ and it follows from the definition of the order of vanishing (note that for small *r*, the values $\gamma (r)$ are near zero) that ${\mathrm {ord}}_0 g_{|f|}\ge {\mathrm {ord}}_0 f$ and so ${\mathrm {ord}}_0 g_{|f|}\ge 2$ as required^13^. $\square$

#### Remark 3.14

The equivalence $(2)\Leftrightarrow (3)$ allows us to extend the Definition 3.9 to any set being the graph of a definable function.

#### Example 3.15

The implication $(2)\Rightarrow (1)$ does not hold in general. To verify this, consider the semi-algebraic function

$$\begin{aligned} f(x,y)={\left\{ \begin{array}{ll} 0, &{}x\le 0\>{\text {or}}\> y\le 0,\\ \frac{y}{x},&{} 0<y\le x\> {\text {and}}\> x^2+y^2>\frac{y^2}{x^2},\\ \left( x^2+y^2\right) \frac{x}{y}, &{} 0<y\le x\> {\text {and}}\> x^2+y^2\le \frac{y^2}{x^2},\\ f(y,x), &{} 0<x<y. \end{array}\right. } \end{aligned}$$

It is easy to check that *f* is continuous. Clearly, $f|_{{\mathbb {R}}_+v}\not \equiv 0$ iff $v\in \mathbb {S}^2\cap \{x,y>0\}=:S$ in which case $f(tv)=t^2(v_1/v_2)$ for $0\le t\le v_2/v_1$ (for greater *t*’s, we get $f(tv)=v_2/v_1$), where $v=(v_1,v_2)$. Hence $s_0(f)=2$.

But if there were ${\mathrm {ord}}_0 f\ge 2$, then we would have in a neighbourhood of zero, $f(x,y)\le C||(x,y)||^2$ for some constant $C>0$. In particular, this would hold for $(x,y)=tv$ for any $v\in S$ and all $t\in (0,\varepsilon )$ with an appropriate $\varepsilon >0$. However, this would lead to $v_1/v_2\le C$ which yields a contradiction when we make $(v_1,v_2)\in S$ tend to (1, 0).

In the next subsection, we will give yet one more characterization of superquadraticity (Proposition 3.18).

#### Remark 3.16

For a given function germ $f:({\mathbb {R}}^n,0)\rightarrow ({\mathbb {R}},0)$, the definition of differentiability at zero gives readily the following two implications:

$$\begin{aligned} f\ {\text {is differentiable at}}\ 0\ {\text {and}}\ \nabla f(0)=0\Rightarrow {\mathrm {ord}}_0 f\ge 1, \end{aligned}$$

and

$$\begin{aligned} {\mathrm {ord}}_0 f\ge 2\Rightarrow \ f\ {\text {is differentiable at}}\ 0 \ {\text {and}}\ \nabla f(0)=0. \end{aligned}$$

The example of $f(x)=|x|^{3/2}$ shows that there may be $f'(0)=0$ and ${\mathrm {ord}}_0 f\in (1,2)$. Note also that without the assumption of definability, the differentiability of *f* at zero together with $\nabla f(0)=0$ need not imply ${\mathrm {ord}}_0 f>1$. We define *f* as follows: consider the sequence $x_\nu :=1/\nu ^{\nu ^2}$ and put $f(x_\nu )=1/\nu ^{\nu ^2+\nu -1}$; then we join the obtained consecutive points by segments in order to obtain a piecewise linear curve defined on (0, 1]. We extend *f* by putting $f(0)=0$ and $f(x)=f(-x)$ for $x\in [-1,0]$. The obtained function is differentiable at zero with $f'(0)=0$. Observe that $f(x_\nu )=\nu x_\nu ^{1+1/\nu }$. If there were constants $r,C,\varepsilon >0$ such that $f(x)\le C|x|^{1+\varepsilon }$ for $|x|<r$, then for all $\nu$ sufficiently large we have $x_\nu <r$ and $1/\nu <\varepsilon$ so that $Cx_\nu ^{1+\varepsilon }<\nu x_\nu ^{1+1/\nu }$ which gives a contradiction. Therefore, ${\mathrm {ord}}_0 f=1$.

### Plane Case

Recall that we call (open) *bi-ball* (or *bidisc* when we are in the plane) *in the direction* $v\in \mathbb {S}^{n-1}$ the open set

$$\begin{aligned} b_v(a,r):=\mathbb {B}(a-rv,r)\cup \mathbb {B}(a+rv,r) \end{aligned}$$

where $$r>0$$.

In this section, we consider a pure one-dimensional closed definable set $X\subset {\mathbb {R}}^2$ with $0\in X$. In particular, in a neighbourhood of zero, *X* consists of finitely many branches ending at zero. Each branch can be realized as the graph of a definable function $f:[0,\varepsilon )\rightarrow {\mathbb {R}}$ with $f(0)=0$ (in properly chosen coordinates in ${\mathbb {R}}^2$).

Observe that the curve $f(t)=at^\alpha +o(t^\alpha )$ (with $a\ne 0$, $\alpha >0$) for $0<t\ll 1$ is ${\mathcal {C}}^1$ at zero iff $\alpha \ge 1$. By analogy to the Definition 3.9, we say that this curve is *superquadratic* at zero iff $\alpha <2$. In particular, if *X* is a graph, it is superquadratic iff one of the two branches is superquadratic. Let us note the following easy lemma.

#### Lemma 3.17

If $\gamma :[0,\varepsilon )\rightarrow [0,+\infty )$ is superquadratic with $\gamma (0)=\gamma '(0)=0$, then for any $r>0$ the disc $D_r:=\mathbb {B}((0,r),r)\subset \{y>0\}$ tangent to the *x*-axis at zero contains points of $\gamma$ inside.

#### Proof

It follows from the obvious observation that if $g:[0,r)\rightarrow {\mathbb {R}}_+$ denotes the usual parametrization of the lower part of the circle $\partial D_r$ through zero, then $g(x)=\frac{1}{2r}x^2+o(x^2)$ near zero. At the same time, $\gamma (x)=ax^\alpha +o(x^\alpha )$ with $a>0$ and $\alpha \in (0,2)$ and so there must be $g(x)<\gamma (x)$ for small *x*. $\square$

This Lemma is an observation that leads to yet another characterization of superquadraticity for any hypersurface $X\subset {\mathbb {R}}^n$:

#### Proposition 3.18

Let $X\subset {\mathbb {R}}^n$ be a closed definable set such that the tangent cone $C_0(X)$ is a linear hyperplane and $X\cap U$ is a graph over it, for some neighbourhood *U* of $0\in X$. Then the following assertions are equivalent:

1. *X* is superquadratic at the origin.
2. For any $r>0$, $b_{\nu (0)}(0,r)\cap X\ne \varnothing$, where $\nu (0)$ is a unit normal to *X* at 0.

#### Proof

Choose coordinates in ${\mathbb {R}}^n={\mathbb {R}}^{n-1}_{x}\times {\mathbb {R}}_{t}$ so that $C_0(X)=\{t=0\}$ and write $X=\Gamma _f$ in a neighbourhood of zero. Fix $\nu (0)=(0,1)$.

We start with $(1)\Rightarrow (2)$. Suppose that for some $r>0$, $b_{\nu (0)}(0,r)\cap X=\varnothing$. This implies that for all $x\in {\mathbb {R}}^{n-1}$ sufficiently close to zero, we have $(x,|f(x)|)\notin \mathbb {B}(r\nu (0),r)$. On the other hand, observe that for $0<||x||< r$ we have $(x,(1/r)||x||^2)\in \mathbb {B}(r\nu (0),r)$. Summing up, in a neighbourhood of zero, $|f(x)|\le (1/r)||x||^2$ which means by Proposition 3.13 that *X* is not superquadratic.

In order to prove $(2)\Rightarrow (1)$, assume that *X* is not superquadratic at zero. Then by Proposition 3.13 we conclude that ${\mathrm {ord}}_0 f\ge 2$, i.e. $|f(x)|\le c||x||^2$ for $||x||<\varepsilon$ where $c,\varepsilon >0$ are constants. Observe that for any $0<r<1/(2c)$, the graph of $t=c||x||^2$ does not enter the ball $\mathbb {B}((0,r),r)$. This readily implies that $X\cap b_{\nu (0)}(0,r)=\varnothing$, provided we have taken $r<\min \{1/(2c),\varepsilon \}$. $\square$

We go back to the plane case.

First, we recall that for a ${\mathcal {C}}^2$ smooth curve $y=f(x)$ its curvature radius at (*x*, *f*(*x*)) is given by $r(x)=1/\kappa (x)$ where 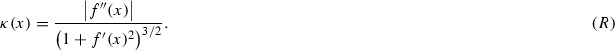 Moreover, it is known that the point (*x*, *f*(*x*)) realizes the distance $d((a,b),\Gamma _f\cap U)$ (where *U* is a small neighbourhood of (*x*, *f*(*x*))^14^) if $v:=(a,b)-(x,f(x))$ is orthogonal to $\Gamma _f$ at (*x*, *f*(*x*)) and $||v||<r(x)$.

#### Theorem 3.19

Assume that $0\in {\mathrm {Reg}}_1 X\cap {\mathrm {Sng}}_2 X$. Then $0\in \overline{M_X}$ iff *X* is superquadratic at the origin.

#### Proof

The problem is local and so we may assume that $X\setminus \{0\}$ is ${\mathcal {C}}^2$-smooth. Furthermore, the tangent cone $C_0(X)$ being a line we may write *X* as the graph of a ${\mathcal {C}}^1$ definable function $f:(-1,1)\rightarrow {\mathbb {R}}$ with $f(0)=f'(0)=0$ and that is ${\mathcal {C}}^2$ outside zero.

Write $\nu (t)=(-f'(t),1)/||(-f'(t),1)||$ for the normal to *X* at (*t*, *f*(*t*)).

We start with proving the ‘if’ part. Let $\Gamma$ be the branch of *f* for $t\ge 0$ and suppose that it is superquadratic. We may also assume that *f* is convex on [0, 1).

Consider $b_{\nu (t)}((t,f(t)),\varepsilon )$, the open bi-disc at (*t*, *f*(*t*)) of radius $\varepsilon$. Since $\Gamma$ is superquadratic at zero, we know by Proposition 3.18 that for any $\varepsilon$, $b_{\nu (0)}((0,0),\varepsilon )\cap \Gamma \ne \varnothing$. Our assumptions imply that it is the upper disc $\mathbb {B}((0,0)+\varepsilon \nu (0),\varepsilon )$ (here $\nu (0)=(0,1)$) that actually intersects $\Gamma$. Therefore, we concentrate our attention only on the ‘upper’ discs that we will make roll to zero from the left.

Denote by $a_\varepsilon (t)=(t,f(t))+\varepsilon \nu (t)$ the centres of the discs for $t\in (-1,0]$. If we start sufficiently far from zero, i.e. for some $t_\varepsilon$ near $-1$, and with a sufficiently small $\varepsilon$, we will have $\mathbb {B}(a_\varepsilon (t_\varepsilon ),\varepsilon )\subset \{(x,y)\mid x<0\}$. In particular,

$$\begin{aligned} d\left( a_\varepsilon (t_\varepsilon ),\Gamma \right) \ge d\left( a_\varepsilon (t_\varepsilon ),\left\{ x\ge 0\right\} \right) >\varepsilon . \end{aligned}$$

Observe that taking a smaller $\varepsilon$ allows us to choose $t_\varepsilon$ nearer to zero.

Consider the continuous function

$$\begin{aligned} \phi _\varepsilon (t)=\varepsilon -d\left( a_\varepsilon (t),\Gamma \right) ,\quad t\in (-1,0]. \end{aligned}$$

We have $\phi _\varepsilon (t_\varepsilon )<0$. On the other hand, since $\mathbb {B}(a_\varepsilon (0),\varepsilon )\cap \Gamma \ne \varnothing$, it is clear that the distance of $a_\varepsilon (0)$ to $\Gamma$ is smaller than the radius of the disc. Hence, $\phi _\varepsilon (0)>0$. Now the Darboux property implies that for some $t'_\varepsilon \in (t_\varepsilon ,0)$ we have $\phi _\varepsilon (t'_\varepsilon )=0$ which means precisely that $\Gamma$ is tangent to the disc $\mathbb {B}(a_\varepsilon (t'_\varepsilon ),\varepsilon )$. So $a_\varepsilon (t'_\varepsilon )$ belongs to the medial axis $M_X$ of the graph of *f* and as $t'_\varepsilon \rightarrow 0$ when $\varepsilon \rightarrow 0$, we conclude that $(0,0)\in \overline{M_X}$.

Of course, in order to roll the disc properly we need an extra assumption. Namely, either the branch $\Gamma '$ of *f* for $t<0$ is concave, or it is non-superquadratic at the origin. Indeed, if it is concave, then due to our choices, the disc rolls on the concave side. If, however, it is convex but non-superquadratic at the origin, then:

- firstly, the definability assures us that $f|_{(-1,0)}$ has a ${\mathcal {C}}^2$ extension through zero, and so we can compute its curvature at any point;
- secondly, it follows from the standard formula for the curvature that the curvature radius in this case stays away from zero.

Summing up, we conclude that for all $\varepsilon$ small enough, the discs of radius $\varepsilon$ are tangent to $\Gamma '$ at any point.

We are left now only with the case when $\Gamma '$ is convex and superquadratic at zero just as $\Gamma$. We proceed as follows.

Any disc $\mathbb {B}((0,\varepsilon ),\varepsilon )$ has points of $\Gamma '$ and $\Gamma$ inside. Fix $\varepsilon$ and keeping the centre at $(0,\varepsilon )$, decrease the radius to $\varepsilon '$ so that $0\notin \overline{\mathbb {B}}((0,\varepsilon ),\varepsilon ')=:B(\varepsilon ')$. Then the intersection of $B(\varepsilon ')$ with the graph of *f* consists of two connected components $C_1,C_2$ the conflict set of which contains a part of $M_X$. Indeed, this is a consequence of Lemma 3.4 and the remark following it. This ends the proof, since we may repeat the argument starting from a smaller $\varepsilon$.

To prove the ‘only if’ part, suppose that *X* is not superquadratic at zero. The function *f* has two branches $f_+(t)=a_+t^{\alpha _+}+o(t^{\alpha _+})$ for $t\in [0,\varepsilon )$ and $f_-(t)=a_-t^{\alpha _-}+o(t^{\alpha _-})$ for $t\in (-\varepsilon ,0]$. Since *X* is not superquadratic, we have $\alpha _+,\alpha _-\ge 2$. In this case, both branches have ${\mathcal {C}}^2$ extensions through zero which means that we are able to roll a ball of a fixed radius on both sides of *X*. This ends the proof. $\square$

#### Example 3.20

In more variables, the situation is not that clear. Consider *X* to be the graph of $f(x,y)=|xy|$. It is easy to see that ${\mathrm {ord}}_0 f\ge 2$, i.e. *f* is not superquadratic at zero, but it is differentiable at this point with $\nabla f(0,0)=0$. However, $M_X$ reaches the origin. Of course, $0\notin {\mathrm {Reg}}_1X$.

Consider two half-lines $\ell _i={\mathbb {R}}_+ v_i$ starting at zero, where $v_i\in \mathbb {S}^1$. We call *bisector* of the angle formed by $\ell _1,\ell _2$ the half-line $\ell ={\mathbb {R}}_+w$ where $w=(v_1+v_2)/2$, provided $w\ne 0$ (i.e. $\angle (\ell _1,\ell _2)\ne \pi$).

For a curve (one-dimensional set) $X\subset {\mathbb {R}}^2$, the tangent cone at any of its points consists of finitely many half-lines. This tangent cone is *linear* if and only if there are only two half-lines $\ell _1,\ell _2$ such that $\angle (\ell _1,\ell _2)=\pi$.

#### Theorem 3.21

Let $X\subset {\mathbb {R}}^2$ be a definable curve with $0\in {{\mathrm {Sng}}}_1 X$. Then $0\in \overline{M_X}$, provided the germ $(X\setminus \{0\},0)$ has at least two connected components.

#### Remark 3.22

By the Nash Lemma (see, e.g. [9, Lemma 1.1]), if $0\in {\mathrm {Reg}}_2 X$, then $M_X$ stays away from *X* in a neighbourhood of the origin. Hence, both Theorems above together with Proposition 3.24 below completely describe the reaching of the singularities in the plane case.

#### Proof

If we restrict to a neighbourhood of zero, then we may assume that $X\setminus \{0\}$ is of class ${\mathcal {C}}^2$ and has a finite number, say *k*, of branches $\Gamma _i$. We know that $k\ge 2$.

First suppose that $k=2$. Then, having a singularity means in this case that $C_0(X)$ is not linear, i.e. it consists of two lines forming an angle different from $\pi$ (but it could be zero). We are working in a ball *B* centred at zero, divided by *X* into two domains $D_1,D_2$ 15. Since $N(0)\subset N_0(X)$, we conclude that on one side of 0 in *B*, say in $D_1$, there are no points whose distance to *X* is realized in zero^16^. Of course, this does not depend on the radius of *B* (provided it is sufficiently small).

Consider now any small ball $B_\varepsilon \subsetneq B$ centred at zero. From the assumptions made so far, we infer that $C_\varepsilon :=D_1\cap \partial B_\varepsilon$ is an arc of the circle $\partial B_\varepsilon$ going from $\Gamma _1$ to $\Gamma _2$ 17. If we restrict to it the function $x\mapsto d(x,\Gamma _1)-d(x,\Gamma _2)$, then we see that it changes sign, whence $C_\varepsilon \cap {\mathrm {Conf}}(\Gamma _1,\Gamma _2)\ne \varnothing$.

If we pick a point $a\in C_\varepsilon \cap {\mathrm {Conf}}(\Gamma _1,\Gamma _2)$, then from the definition of the conflict set, it follows that here are two points $b_i\in \Gamma _i$ such that $||a-b_1||=d(a,\Gamma _1)=d(a,\Gamma _2)=||a-b_2||$. But $X=\Gamma _1\cup \Gamma _2$ and $0\notin m(a)$, whence $b_1,b_2\in m(a)$ and so $a\in M_X$ (see also Remark 3.23 hereafter). Since $\varepsilon$ is arbitrarily small, the proof is accomplished.

Now, assume that $k\ge 3$. Actually, the argument is quite the same, the only difference being that the linearity or nonlinearity of the tangent cone changes the start-point. Indeed, if $C_0(X)$ is nonlinear, then we can extract, so to say, two different branches $\Gamma _i, \Gamma _j$ such that

- their tangent half-lines at zero form an angle different from $\pi$;
- in one of the two regions they divide *B* in, call it *D*, there are no other branches of *X*.

Then we can repeat the same argument showing that part of the conflict set ${\mathrm {Conf}}(\Gamma _i,\Gamma _j)$ in *D* is contained in $M_X$ and so it approaches zero.

Otherwise, if $C_0(X)$ is linear, then, again, among the $k\ge 3$ branches of $X\setminus \{0\}$ there must be two branches that satisfy the two requirements above. The only difference is that now we know that the angle between the tangent half-lines is 0, which does not affect the argument.

This ends the proof. $\square$

#### Remark 3.23

Note that in general, for two distinct closed sets *X*, *Y* with a unique common point $X\cap Y=\{a\}$, there is

$$\begin{aligned} M_{X\cup Y}\setminus \left( {\mathrm {Terr}}^o(X)\cup {\mathrm {Terr}}^o(Y)\right) ={{\mathrm {Conf}}}(X,Y)\setminus C(X,Y), \end{aligned}$$

where ${\mathrm {Terr}}^o(X)=\{x\in {\mathbb {R}}^n\mid d(x,X)<d(x,Y)\}$ is the *open territory* of *X* and $C(X,Y)=\{x\in {\mathrm {Conf}}(X,Y)\mid m_X(x)=m_Y(x)\}$. The inclusion ‘$\supset$’ follows from the proof above: if *x* is equidistant to *X* and *Y* (so that *x* does not belong to any of the open territories) but $m_X(x)\ne m_Y(x)$, then necessarily $\#m_{X\cup Y}(x)>1$. To see ‘$\subset$’, pick a point *x* in the set on the left-hand side. Then it is equidistant to *X* and *Y* so that it belongs to the conflict set. But $m_X(x)=m_Y(x)$ implies that this set is included in $X\cap Y=\{a\}$, and so $m_{X\cup Y}(x)=\{a\}$, contrary to the assumptions.

By throwing away the open territories of *X* and *Y* from the medial axis, we make sure there are no ‘self-conflict’ parts left. On the other hand, getting rid of *C*(*X*, *Y*) from the conflict set is important in view of the example given in Remark 2.15.

If the intersection $X\cap Y$ has more than one point, there is no such a simple relation between the medial axis and the conflict set as we can see, for instance, from the example of *X* being the unit circle in ${\mathbb {R}}^2$ together with the point (2, 0) and *Y* just the unit circle.

#### Proposition 3.24

Assume that $X\subset {\mathbb {R}}^2$ is a definable curve such that $0\in X$ and the germ $(X\setminus \{0\},0)$ is connected. Then $0\notin \overline{M_X}$.

#### Proof

The tangent cone $C_0(X)$ is a half-line that we can assume to be the *x*-axis for $x\ge 0$. Moreover, we can assume that *X* near zero is a graph of a definable, convex function $f:[0,\varepsilon )\rightarrow {\mathbb {R}}$, $f(0)=0$ that is ${\mathcal {C}}^2$ on $(0,\varepsilon )$ and has a ${\mathcal {C}}^1$ extension through zero with $f'(0)=0$. If the extension can be made of class ${\mathcal {C}}^2$, then by the Nash Lemma we conclude that $0\notin \overline{M_X}$. Suppose thus that there is only a ${\mathcal {C}}^1$ extension. This means that when we write $f(t)=ct^\alpha +o(t^\alpha )$ with $c,\alpha >0$, there must be $\alpha \in (1,2)$, i.e. *f* is superquadratic.

Observe that $N_0(X)=\{(x,y)\mid x\le 0\}$. By the superquadraticity and the convexity of *f*, we conclude that no point (0, *y*) with $y>0$ has its distance to *X* realized at zero. On the other hand, it is obvious that for all other points from $N_0(X)$ sufficiently near zero, the origin is the unique closest point in *X*.

Take a point $a(y)=(0,y)$ with a small $y>0$ and consider the supporting sphere $\mathbb {S}(a(y),d(a(y),X))$. This circle touches *X* in a finite number of points none of which is the origin, by superquadraticity. But the function $y\mapsto \#m(a(y))$ is definable (cf. [6]) which implies that for all $y>0$ near zero it must be constant, equal to 1 (cf. the points in *m*(*a*(*y*)) tend to zero when $y\rightarrow 0^+$ and *X* is a graph near zero). This ends the proof. $\square$

We end this section by describing the tangent cone of $M_X$ when this set reaches *X*. Note that as $X\setminus \{0\}$ consists of finitely many branches ending at zero, the tangent cone $C_0(X)$ is the union of finitely many half-lines being the tangents of these branches. As can be expected, it turns out that the tangent cone of the medial axis consists of all the bisectors of the half-lines forming up $C_0(X)$. However, as the next example shows, not all the pairs of half-lines are to be taken into account.

#### Example 3.25

Let $X\subset {\mathbb {R}}^2$ be the graph of $y=x^2$ together with the curve $y=-x^2$ for $x\ge 0$. Therefore, *X* has three branches ending at the origin and $C_0(X)$ is the *x*-axis. Clearly, $0\in \overline{M_X}$ (as $M_X=(0,+\infty )\times \{0\}$), but only the two branches $y=\pm x^2$ contribute to this. Both have $[0,+\infty )\times \{0\}$ as the tangent cone at zero and so the bisector coincides with this half-line. Of course, it is $C_0(M_X)$.

A similar situation occurs for a $\lambda$-shaped curve.

Before we state and prove the theorem on the tangent cone, let us introduce some notions that will help to state the result.

For a plane definable germ (*X*, 0), we say that two branches $\Gamma _1,\Gamma _2$ at zero are *consecutive*, if they bound a region near zero which does not contain any other branch of *X* 18. There is only one such region, if *X* has more than two branches. In this case, we denote it by $D(\Gamma _1,\Gamma _2)$.

Assume that $0\in \overline{M_X}$. Of course, $M_X$ is the union of all the medial axes $M_{\Gamma _1\cup \Gamma _2}\cap D(\Gamma _1,\Gamma _2)$ for all pairs of consecutive branches $\Gamma _1,\Gamma _2$ (assuming that *X* has more than two branches). We say that a region delimited by a pair of consecutive branches *contributes to* $M_X$ *at zero*, if the origin belongs to the closure of the corresponding medial axis^19^.

If *X* has only two branches at zero, then there are two regions delimited by them, which we denote $D_\pm$. It may happen that $M_X$ appears in only one of these regions (as for $X:y^2=x^3$), or in both of them (as for $X:y={\mathrm {sgn}} x |x|^{3/2}$). In this case it is natural to consider the two branches as two different pairs of consecutive branches, distinguished by their ordering.

Finally, let us recall one theorem from [1] that we shall use in the proof.

#### Theorem 3.26

(Birbrair–Siersma) Let $X_1,\dots ,X_k\subset {\mathbb {R}}^n$ be closed, definable, pairwise disjoint, nonempty sets such that 0 belongs to $K:={\mathrm {Conf}}(X_1,\dots , X_k)$ and let $S:=\mathbb {S}(0,d(0,X_1))$ be the supporting sphere at 0.

Then $C_0(K)$ is the cone spanned over the conflict set ${\mathrm {Conf}}_S(\tilde{X}_1,\dots ,\tilde{X}_k)$ in the sphere, where $\tilde{X}_i:=X_i\cap S$.

Here, what we mean by a *cone spanned over* a subset *E* of the sphere *S* centred at zero is the set $\bigcup \{{\mathbb {R}}_+ v\mid v\in E\}$. The conflict set *in the sphere* is computed with respect to the geodesic metric in *S* (cf. Remark 2.15).

#### Theorem 3.27

Assume that $0\in \overline{M_X}\cap X$ where *X* is a pure one-dimensional closed definable set in the plane. Then $C_0(M_X)$ is the union of the bisectors of all the pairs of half-lines forming up $C_0(X)$ and given by pairs of branches delimiting regions that contribute to $M_X$ at zero.

#### Proof

Since $M_X$ is nowheredense, definable and $0\in \overline{M_X}\setminus M_X$, it follows from the Curve Selection Lemma that $\dim _0 M_X=1$. Therefore, the germ of $M_X$ at zero consists of finitely many branches (arcs) $M_i$ ending at the origin. Each of them gives a half-line, and the union of these half-lines forms the tangent cone $C_0(M_X)$.

Of course, each branch $M_i$ must lie between exactly two consecutive branches $\Gamma _{i,1},\Gamma _{i,2}$ of *X*. Moreover, by the triangle inequality, in the region delimited by two branches $\Gamma _1,\Gamma _2$ of *X*, there cannot be more than one branch $M_i$ 20.

Therefore, we may restrict our considerations to the case when *X* has only two branches $\Gamma _1,\Gamma _2$, both of class ${\mathcal {C}}^1$ 21, delimiting a region *D* in which the medial axis is a curve *M* ending at zero. From the definability, it follows that *M* may be assumed to be a ${\mathcal {C}}^1$ curve and $C_0(M)$ is the tangent half-line at $0\in \overline{M}$.

From [9] Theorem 4.13, we know that apart from a finite set of points in *M*, we have $\dim m(x)=0$ along *M*. Moreover, as *m*(*x*) varies continuously when $M\ni x\rightarrow 0$, we conclude that $\#m(x)=2$ for $x\in M$, for the points form rails on which the disc $\mathbb {B}(x,d(x,X))$ rolls to zero, and so the rails must coincide with the two branches^22^.

In view of our previous results, the assumption $0\in \overline{M_X}$ means that either $0\in {{\mathrm {Sng}}}_1 X$, or *X* is superquadratic at zero. In both cases, it follows that no point in *D* has its distance to *X* realized in zero (cf. the previous proofs).

Therefore, if we parametrize *M* by $\gamma :(0,\varepsilon )\rightarrow M$ with $\gamma (t)\rightarrow 0$ when $t\rightarrow 0$, we will have $C_0(M)={\mathbb {R}}_+\gamma _+'(0)$. Moreover, for each $t\in (0,\varepsilon )$, there are two points $\eta _i(t)\in \Gamma _i$ realizing $d(\gamma (t),X)$ and $\eta _i(t)\rightarrow 0$ when $t\rightarrow 0$. Observe that the tangents to $\Gamma _i$ at $\eta _i(t)$ tend to the tangent direction of $\Gamma _i$ at zero.

Since $\#m(\gamma (t))=2$, we can invoke Lemma 3.3 to see that *M* near $\gamma (t)$ coincides with the conflict set of the two branches. Thus, we are in a position that allow us to use the Birbrair–Siersma Theorem: the conflict set of the two points $\eta _i(t)$ in the circle $\mathbb {S}(\gamma (t),d(\gamma (t),X))$ consists obviously of two antipodal points, since the cone spanned over them is the tangent cone $C_{\gamma (t)}(M)$ which must coincide with the tangent to *M* at $\gamma (t)$. Still more, this line is clearly the bisector of the angle formed by the tangents to the circle at the points $\eta _i(t)$ (note that they cannot be parallel).

Of course, when *t* tends to zero, the tangent direction $C_{\gamma (t)}(M)$ approaches the tangent direction to *M* at zero. It follows that $C_0(M)$ must be the bisector of the tangent half-lines of the branches, as required. $\square$

#### Example 3.28

In the non-definable setting, the tangent cone $C_0(M_X)$ (when $M_X$ reaches the set *X* at zero) may be quite big. To ascertain this, consider $X=\{0\}\cup \bigcup _{\nu =1}^{+\infty }\{(x_\nu ,0)\}\subset {\mathbb {R}}^2$ where $x_\nu =1/\nu$. Then

$$\begin{aligned} M_X=\bigcup _{\nu =1}^{+\infty } \left\{ \frac{x_\nu +x_{\nu +1}}{2}\right\} \times {\mathbb {R}} \end{aligned}$$

and so $0\in \overline{M_X}$, but $C_0(M_X)=\{(x,y)\mid x\ge 0\}$, while $C_0(X)=[0,+\infty )\times \{0\}$.

#### Example 3.29

Let $X=\{(x,x^2)\mid x\in [0,1)\}\cup \{(x,x^3)\mid x\in [0,1)\}$. Then $M_X$ near zero is clearly a curve lying between the two branches of $X\setminus \{0\}$. Since these branches have a common tangent $[0,+\infty )\times \{0\}$ at zero, this line is also the tangent cone of $M_X$ at the origin.

From the last Theorem, we obtain a kind of symmetry property of plane analytic curves.

#### Corollary 3.30

Let $X\subset {\mathbb {R}}^2$ be a real-analytic curve germ at zero and such that $X\setminus \{0\}$ consists of only two branches and $0\in \overline{M_X}$. Then in a neighbourhood *U* of zero, the medial axis $M_X$ is a half-line that is a symmetry axis of $X\cap U$.

#### Proof

In view of the preceding results, there are two possibilities^23^: either $0\in {{\mathrm {Sng}}}_1X$, or $0\in {\mathrm {Reg}}_1X\cap {{\mathrm {Sng}}}_2 X$ with *X* superquadratic at the origin. In the first case, by [14] Corollary 5.6, we know that $C_0(X)$ is a half-line $\ell$, that we may assume to be $\{0\}\times [0,+\infty )$, whereas in the second one, it is a line *L* that we assume to be the *x*-axis. Using the definition of the tangent cone, we may assume in both cases that in a neighbourhood of the origin *X* is a graph over an interval $(-\varepsilon ,\varepsilon )$ in the *x*-axis. Consider $F=0$ to be an analytic equation of *X* in the same neighbourhood.

Let *h* be the branch over $(-\varepsilon ,0]$ and *g* the branch over $[0,\varepsilon )$. They both are ${\mathcal {C}}^1$ at zero and due to the Puiseux Theorem, for some integer $p>0$, $g(t^p)$ has an analytic extension through zero onto $(-\delta ,\delta )$ for some $\delta \in (0,\varepsilon )$. Then, we obtain

$$\begin{aligned} F(s,g(s))\equiv 0,\quad s\in [0, \root p \of {\delta }). \end{aligned}$$

Therefore, by the identity principle, this holds true for $|s|<\root p \of {\delta }$. But we may repeat the same argument with *h* and so we conclude that $g(-s)=h(s)$ for $s\in (0,\delta )$ (if $\delta$ was chosen $<1$) which gives the symmetry sought after (since the germ of $M_X$ at zero depends only on the germ of *X* at this point) and proves that $M_X$ is a half-line near zero, as well. $\square$

#### Remark 3.31

It follows from this Corollary that, for instance, the superquadratic curve $y={\mathrm {sgn}}(x)|x|^{3/2}$ is not analytic at the origin.

## Hypersurfaces in ${\mathbb {R}}^n$

### Tangent Cone Method at ${\mathcal {C}}^1$-Singular Points

We begin with a not so obvious proof of an obvious fact:

#### Proposition 4.1

Let $V\subset {\mathbb {R}}^n$ be a closed real cone with vertex at the origin and such that

1. $${\mathrm {int}} V=\varnothing $$;
2. *V* is not contained in a hyperplane.

Then $$0\in \overline{M_V}$$.

#### Proof

It is sufficient to prove that $C_V\ne \varnothing$ 24 since, firstly, $\overline{C_V}=\overline{M_V}$, and secondly, picking a point $x\in C_V$, we may move the maximal ball $\mathbb {B}(x,d(x,V))$ towards the vertex by homothety and it stays maximal.

By (1), there is a point $x\in {\mathbb {R}}^n\setminus V$. If $x\notin C_V$, then we have exactly one closest point $m(x)\in V$. Now, we consider the balls

$$\begin{aligned} B_t(x):=\mathbb {B}\left( m(x)+t(x-m(x)),td(x,V)\right) , \quad t\ge 1 \end{aligned}$$

whose closure touch *V* at *m*(*x*). Let

$$\begin{aligned} t(x)=\sup \left\{ t\ge 1\mid B_t(x)\subset {\mathbb {R}}^n\setminus V\right\} . \end{aligned}$$

Either *t*(*x*) is finite, in which case the corresponding centre belongs to $C_V$, or $t(x)=+\infty$ which means that *V* does not enter the open half-space $H_x:=\{y\in {\mathbb {R}}^n\mid \langle y, x-m(x)\rangle >0\}$ when translated to *m*(*x*). But as *V* is a cone, we have $H_x\cap V=\varnothing$. If the latter occurs we say that $H_x$ is a *supporting* half-space for *V*.

Any open half-space is defined by a unique unit vector $v\in \mathbb {S}^{n-1}$ through the inequality $\langle y,v\rangle >0$, and we denote this half-space by $H_v$.

For the rest of the proof, let us suppose that $C_V=\varnothing$. Then by the preceding observation, the set

$$\begin{aligned} P=\left\{ v\in \mathbb {S}^{n-1}\mid H_v\ {\text {is a supporting half-space for}}\ V\right\} \end{aligned}$$

is nonempty, and

$$\begin{aligned} V\subset \bigcap _{v\in P} H_v'=:W \end{aligned}$$

where $H'_v=\{y\in {\mathbb {R}}^n\mid \langle y,v\rangle \le 0\}$ is the complement of $H_v$. If we had $V\ne W$, then we would find a point $w\in W\setminus V$. It would readily imply that *w* is the centre of a maximal ball contrary to the assumptions. This is so because, by the assumptions, *w* has a unique closest point *m*(*w*), and so for $v:=(w-m(w))/||w-m(w)||$, $H_v$ is a supporting half-space for *V*, whence $V\subset H'_v$, i.e. $v\in P$. But, clearly, $\langle w-m(w), m(w)\rangle =0$, and so we can write

$$\begin{aligned} \langle w, w-m(w)\rangle =\langle w, w-m(w)\rangle -\langle m(w), w-m(w)\rangle =\left| \left| w-m(w)\right| \right| ^2>0 \end{aligned}$$

which means that $w\in H_v$, contrary to the choice of *w*.

Hence $V=\bigcap _{v\in P} H'_v$. This, however, contradicts the conjunction of (1) and (2).

Indeed, we have just shown that *V* is a convex cone. Let *k* denote the maximal number of linearly independent vectors in $V\cap \mathbb {S}^{n-1}$. If $k=n$, then *V* must contain an *n*-dimensional simplex (formed by the *n* points on the sphere and the origin) and so has nonempty interior. Otherwise, if $k<n$, it follows that *V* is contained in a hypersurface. $\square$

#### Remark 4.2

By homothety, we easily see that for any closed cone *V* with vertex at *a*, $\overline{M_V}$ is also a cone with *a* as the vertex.

Observe also that assumption (2) implies that *V* is nonlinear, i.e. not a vector subspace.

We need both assumptions in the Proposition above:

#### Example 4.3

Consider $V_1=\{(x,y)\in {\mathbb {R}}^2\mid y\ge |x|\}$; it has nonempty interior. Clearly, $M_{V_1}=\varnothing$.

Let $V_2=[0,+\infty )\times \{0\}\subset {\mathbb {R}}^2$. Then $V_2$ is contained in a line of the plane and $M_{V_2}=\varnothing$.

It follows from the definition of the tangent cone $C_0(X)$ that $v\in C_0(X)\setminus \{0\}$ iff for any $\varepsilon ,\delta >0$,

$$\begin{aligned} X\cap \mathbb {B}(0,\varepsilon )\cap V(v,\delta )\ne \varnothing \end{aligned}$$

where $$V(v,\delta )=\{tw\mid t>0, w\in {\mathbb {R}}^n\setminus \{0\}:||(v/||v||)-(w/||w||)||<\delta \}$$.

In particular, if we assume that $C_0(X)$ satisfies the assumptions of the previous Proposition, we see that, obviously, $0\in {{\mathrm {Sng}}}_1 X$, and it is rather not astonishing that also $0\in \overline{M_X}$, as we will show in the next Theorem. Moreover, we could expect that then $C_0(M_X)=\overline{M_{C_0(X)}}$.

The general idea is clear: we obtain the tangent cone by looking at *X* in the point 0 through a strong magnifying glass so that it becomes a set of half-lines; it is thence natural to expect that near 0 the medial axis of *X* should behave like the medial axis of the cone itself.

#### Remark 4.4

Note that this does not solve the problem $0\in {{\mathrm {Sng}}}_1 X$ entirely since we do not consider the cases when $C_0(X)$ is linear (like for the graph of $z=y|x|$) or degenerated (like for the horn $x^2+y^2=z^3$).

Not only this remark should stop our optimism, but also the following example:

#### Example 4.5

We can hardly expect equality: let *X* be the union of the horn $x^2+y^2=z^3$ together with $z=-||(x,y)||$. Then $V:=C_0(X)$ consists of $x=y=0,z\ge 0$ together with $z=-||(x,y)||$ and so satisfies the assumptions of Proposition 4.1, but $M_X$ is the *z*-axis without the origin, so that $C_0(M_X)$ is the *z*-axis, whereas $M_V$ is just $x=y=0,z<0$ which means that $C_0(M_V)$ is only $x=y=0,z\le 0$.

In view of this example, the best result we can have is the following one obtained using the main result of [10]:

#### Theorem 4.6

Assume that $X\subset {\mathbb {R}}^n$ is a definable hypersurface with tangent cone $V=C_0(X)$ satisfying the assumptions of the Proposition 4.1. Then $0\in \overline{M_X}$ and $C_0(M_X)\supset \overline{M_V}$.

#### Proof

As we are in the definable case, we know that *V* is the Kuratowski limit when $t\rightarrow 0^+$ of the dilatations (1 / *t*)*X*, $t>0$ (this follows from the Curve Selection Lemma). Hence by [10],

$$\begin{aligned} M_V\subset \liminf _{t\rightarrow 0^+} M_{(1/t)X}. \end{aligned}$$

But it is easy to see that $M_{(1/t)X}=(1/t)M_X$ (by homothety) and so the limit inferior is actually a limit and coincides with $C_0(M_X)$. Finally, as observed in [12], for a definable set we have $C_0(E)=\lim _{t\rightarrow 0^+}(1/t) E$ also in the case when $0\notin \overline{E}$ in which case the limit is empty. Therefore, since we know by Proposition 4.1 that $0\in \overline{M_V}$, we obtain the result sought for. $\square$

#### Remark 4.7

It is possible to give a straightforward proof which, however, requires rather technical computations.

### The Medial Axis Near ${\mathcal {C}}^1$-Smooth Points of Hypersurfaces

In this section, we assume that $X={\mathrm {Reg}}_1 X$ is a ${\mathcal {C}}^1$ codimension 1 submanifold. Then locally we can define at each point $a\in X$ a continuous unit normal vector field (‘exterior’ or ‘interior’ depending on the side of *X* we choose) $U\cap X\ni x\mapsto \nu (x)\in \mathbb {S}^{n-1}$, where *U* is an appropriate neighbourhood of *a* such that $U\cap X$ is connected and is the graph of a ${\mathcal {C}}^1$ function. We call such a neighbourhood *admissible*. Then we consider

$$\begin{aligned} L_a(U):=\inf \left\{ L>0 \mid \left| \left| \nu (x)-\nu (x')\right| \right| \le L\left| \left| x-x'\right| \right| \mid x,x'\in U\cap X\right\} . \end{aligned}$$

Recall that $\inf \varnothing =+\infty$ so that we have $L_a(U)\in [0,+\infty ]$; $L_a(U)$ is a Lipschitz constant for $\nu$.

Let us note that for a smaller neighbourhood $a\in U'\subset U$, there is $L_a(U')\le L_a(U)$ so that if $L_a(U)$ is finite, it remains finite for a basis of neighbourhoods of *a*.

#### Remark 4.8

If the neighbourhood *U* is of the form $V\times W\subset {\mathbb {R}}^{n-1}\times {\mathbb {R}}$ (with appropriately chosen coordinates) so that $U\cap X$ is the graph $\Gamma _f$ of a ${\mathcal {C}}^1$ function $f:V\rightarrow W$, then if *f* is of class ${\mathcal {C}}^{1,1}$ 25, we have $L_a(U)<+\infty$ 26.

#### Proposition 4.9

Assume that $L_a(U)<+\infty$ and let $x\in U$ be such that $m(x)\subset U$. If $d(x)<1/L_a(U)$, then $\#m(x)=1$.

#### Proof

We may assume $x\notin X$, so that $d(x)>0$. Take $y,y'\in m(x)$ and observe that we necessarily have $x=y\pm d(x)\nu (y)=y'\pm d(x)\nu (y')$ with the same sign in both cases, for by assumption $U\cap X$ is a graph. Hence $||y-y'||=d(x)||\nu (y)-\nu (y')||$ and so $y\ne y'$ would imply $L_a(U)\ge 1/d(x)$ contrary to the assumption. $\square$

From this, we immediately obtain

#### Corollary 4.10

If $L_a(U)<+\infty$ (for some admissible *U*), then $a\notin \overline{M_X}$.

#### Proof

It is sufficient to note that for $x\in V\subset U$ where *V* is a sufficiently small neighbourhood of *a*, we have $m(x)\subset U\cap X$ by Proposition 2.17. Hence, the preceding Proposition applies to any point from $W=\mathbb {B}(a,1/L_a(U))\cap V$. Hence, $m|_W$ is univalued. $\square$

For $x\in X$, we extend the definition of an open bi-ball

$$\begin{aligned} b(x,r)=\mathbb {B}(x+r\nu (x),r)\cup \mathbb {B}(x-r\nu (x),r) \end{aligned}$$

tangent to $T_xX$ by putting $b(x,0):=\varnothing$.

#### Definition 4.11

We call

$$\begin{aligned} r_a(U):=\sup \left\{ r\ge 0\mid b(x,r)\cap X=\varnothing , x\in U\cap X\right\} \end{aligned}$$

the *local (double) reach radius of* *X* *in* *U*.

#### Remark 4.12

Let us observe that for a neighbourhood $a\in U'\subset U$, there is $r_a(U')\ge r_a(U)$. Note also that $r_a(U)=+\infty$ for some *U* iff *X* is contained in an affine hyperplane (and then $r_a(U')=+\infty$ for any neighbourhood $U'$ of *a*). It is important to stress that we are looking at the intersection of the biballs with the whole of *X*, not only with $U\cap X$.

#### Proposition 4.13

$a\in \overline{M_X}$ implies $r_a(U)=0$ for all sufficiently small neighbourhoods $U\ni a$.

#### Proof

Observe that if $r_a(U)>0$ for some *U*, then for any ball $\mathbb {B}(a,2\rho )\subset U$ with $2\rho <r_a(U)$ each point $x\in \mathbb {B}(a,\rho )$ has its distance to *X* realized in $\mathbb {B}(a,2\rho )\cap X$. Then we have necessarily $\#m(x)=1$ and so $\mathbb {B}(a,2\rho )\cap M_X=\varnothing$. $\square$

The local reach radius is closely related to $L_a(U)$, provided it is bounded and its boundedness comes from the shape of *X* near *a* and not from other parts of *X*. To be more precise, the following Theorem 4.16 does not hold for $X={\mathbb {R}}\times \{-1,1\}$ since $r_a(U)=1$, but $L_a(U)=0$ at all points and for all admissible *U*. Therefore, we introduce one more notion:

#### Definition 4.14

We say that the local reach radius of *X* is *intrinsically bounded at a*, if for some admissible neighbourhood *U* of *a* there is $r_a(U)<+\infty$ and for any $r>r_a(U)$, we can find $r>r'>r_a(U)$ and a point $b\in U\cap X$ such that $\varnothing \ne b(b,r')\cap X\subset U$.

#### Remark 4.15

Essentially, this means that the germ of $M_X$ at *a* depends only on the germ of *X* at *a*. The admissibility of *U* is important in view of the example preceding Definition 4.11.

#### Theorem 4.16

Let *X* be a ${\mathcal {C}}^1$ hypersurface and *U* an admissible neighbourhood of a point $a\in X$. Then $r_a(U)\le 1/L_a(U)$ (with the convention that $1/0:=+\infty$). If, moreover, $r_a(U)$ is intrinsically bounded, then, $r_a(U)=1/L_a(U)$ for a sufficiently small neighbourhood $U\ni a$.

#### Proof

Take $r_a(U)>r>0$ and write $b_{\pm }=b\pm r\nu (b)$, $c_{\pm }=c\pm r\nu (c)$ where $b\ne c$ are two points in $U\cap X$. Clearly,

$$\begin{aligned} \left| \left| b_+-c_-\right| \right| \ge 2r, \end{aligned}$$

as the points in question lie on different sides of *X* which means that $\mathbb {B}(b_+, r)\cap \mathbb {B}(c_-,r)=\varnothing$. Squaring both sides we can rewrite this inequality as 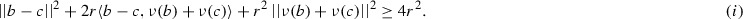 The same kind of computation leads from $||b_--c_+||\ge 2r$ to 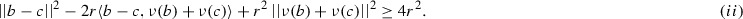 Note that

$$\begin{aligned} \left| \left| \nu (b)+\nu (c)\right| \right| ^2=\left| \left| \nu (b)\right| \right| ^2 +\left| \left| \nu (c)\right| \right| ^2+2\langle \nu (b),\nu (c)\rangle \end{aligned}$$

and $||\nu (b)||=||\nu (c)||=1$. Therefore, $((i)+(ii))/2$ yields

$$\begin{aligned}&\left| \left| b-c\right| \right| ^2+r^2(2+2\langle \nu (b),\nu (c)\rangle )\ge 4r^2\\&\quad \left| \left| b-c\right| \right| ^2\ge 2r^2\left( 1-\langle \nu (b),\nu (c)\rangle \right) =r^2\left| \left| \nu (b)-\nu (c)\right| \right| ^2. \end{aligned}$$

This implies that $L_a(U)\le 1/r$ and so

$$\begin{aligned} r_a(U)\le 1/L_a(U). \end{aligned}$$

Now, suppose that $r_a(U)$ is intrinsically bounded and there is no equality, so that we can find

$$\begin{aligned} 1/L_a(U)>r>r_a(U). \end{aligned}$$

In order to obtain a contradiction, we argue as follows. By the intrinsic boundedness, taking a smaller *r* if necessary, we can find a point $b\in U\cap X$ such that $\varnothing \ne b(b,r)\cap X\subset U$ 27. There is a point *c* in this intersection, say, that $c\in \mathbb {B}(b_+,r)\cap X$, such that $||b_+-c||=d(b_+,X)$. But this distance is smaller than $r<1/L_a$, hence by Proposition 4.9 28, we have $m(b_+)=\{c\}$. Moreover,

$$\begin{aligned}{}[b,b_+]\cap N(b)=[b,u]\subsetneq [b,b_+] \end{aligned}$$

for some *u*. In particular, $b\in m(u)$ and for any $x\in (u,b_+]$, $b\notin m(x)$.

Take a sequence $(u,b_+]\ni x_\nu \rightarrow u$. Then $\limsup _{\nu \rightarrow +\infty } m(x_\nu )\subset m(u)$ (cf. Proposition 2.17), whence we can find a sequence $m(x_\nu )\ni y_\nu \rightarrow y_0\in m(u)$. But $d(x_\nu ,X)$ converges both to $d(u,X)=||u-b||$ and to $||u-y_0||$ and so the latter is strictly smaller than $r<1/L_a(U)$. Proposition 4.9 implies that $y_0=b$ and $m(u)=\{b\}$.

We have $x_\nu =b+||x_\nu -b||\nu (b)=y_\nu +||x_\nu -y_\nu ||\nu (y_\nu )$. Let us denote

$$\begin{aligned} \alpha _\nu :=\angle (y_\nu -b,x_\nu -b),\quad \beta _\nu :=\angle \left( b-x_\nu ,y_\nu -x_\nu \right) . \end{aligned}$$

Since *X* is ${\mathcal {C}}^1$, we have $\nu (y_\nu )\rightarrow \nu (b)$ and so $\alpha _\nu \rightarrow \pi /2$ and $\beta _\nu \rightarrow 0$. By the Sine Law,

$$\begin{aligned} \frac{\sin \alpha _\nu }{\left| \left| y_\nu -x_\nu \right| \right| }=\frac{\sin \beta _\nu }{\left| \left| y_\nu -b\right| \right| }. \end{aligned}$$

This leads to 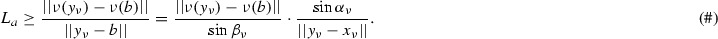 It remains to observe that, passing to the limit, on the one hand we get

$$\begin{aligned} \frac{\sin \alpha _\nu }{\left| \left| y_\nu -x_\nu \right| \right| }\rightarrow \frac{1}{\left| \left| b-u\right| \right| }>\frac{1}{r}>L_a \end{aligned}$$

where the first inequality comes from the fact that $u\in (b,b_+)$ and $||b-b_+||=r$, while on the other one,

$$\begin{aligned} \frac{\left| \left| \nu (y_\nu )-\nu (b)\right| \right| }{\sin \beta _\nu }\rightarrow 1, \end{aligned}$$

because

$$\begin{aligned} \frac{\left| \left| \nu (y_\nu )-\nu (b)\right| \right| ^2}{\sin ^2 \beta _\nu }&=2\frac{1-\langle \nu (y_\nu ),\nu (b)\rangle }{1-\cos ^2\beta _\nu }\\&=2\frac{1-\cos \angle \left( \nu (y_\nu ),\nu (b)\right) }{1-\cos ^2\angle \left( \nu (y_\nu ),\nu (b)\right) }\\&=2\frac{1}{1+\cos \angle \left( \nu (y_\nu ),\nu (b)\right) }. \end{aligned}$$

Summing up, $(\#)$ leads to a contradiction as wanted. $\square$

#### Remark 4.17

Up to now we have had no need for definability. If we suppose that $X=\Gamma _f$ is definable, it readily implies that for $\tilde{\nu }(x)=(-\nabla f(x),1)/||(-\nabla f(x),1)||$ the set

$$\begin{aligned} E:=&\left\{ (r,L)\in (0,1)\times (0,+\infty )\mid \forall x,x'\in \mathbb {B}_{n-1}(a,r),\right. \\&\left. \left| \left| \tilde{\nu }(x)-\tilde{\nu }(x')\right| \right| \le L\left| \left| (x,f(x))-(x',f(x'))\right| \right| \right\} \end{aligned}$$

is definable. Put $\varphi (r):=\inf E_r$ where $E_r$ is the *r*-section of *E* and let $\pi (r,L)=r$. Then $\varphi (r)=+\infty$ iff $r\notin \pi (E)$ which implies that $\varphi :(0,1)\rightarrow [0,+\infty ]$ is a definable function (cf. [10, Lemma 4.4]). This in turn means that $\varphi$ has a well-defined limit $L_a:=\lim _{r\rightarrow 0^+}\varphi (r)$ 29. According to the remark following the definition of $L_a(U)$, $\varphi$ is an increasing function, so that $L_a=\inf _{r\in (0,1)} \varphi (r)$.

#### Remark 4.18

By Proposition 3.18, if *X* is superquadratic at *a*, then $r_a(U)=0$ for any neighbourhood *U* of *a*. The converse is not true as we can see from the definable example of the graph of the ${\mathcal {C}}^1$ function $f(x,y)=y|x|^{3/2}$. Here, ${\mathrm {ord}}_0 f\ge 2$, so that *f* is not superquadratic at the origin, but at any point along the *y*-axis (apart from zero), *f* is superquadratic whence $r_0(U)=0$ for any neighbourhood *U* of zero.

We can complete the discussion by the following result.

#### Theorem 4.19

For a hypersurface *X* with $a\in {\mathrm {Reg}}_1 X$, the following conditions are equivalent:

1. for some neighbourhood *U* of *a*, there is $r_a(U)>0$;
2. for some admissible neighbourhood *U* of *a* and some $\varepsilon >0$, the segments $\nu _x^\varepsilon :=[x-\varepsilon \nu (x), x+\varepsilon \nu (x)]$, $x\in U\cap X$, are pairwise disjoint, where $\nu$ is a local unit normal field on $U\cap X$;
3. $$a\notin \overline{M_X}$$.

#### Proof

$(1)\Rightarrow (3), (2)$. Since $r_a(U)>0$, by Proposition 4.13 we conclude that $a\notin \overline{M_X}$ which gives (3). In particular, in some neighbourhood $V\subset U$ of *a*, *m* is univalued, and we may assume that $m(V)\subset U$. Take $0<\varepsilon <r_a(U)$ such that for any $x\in V'\cap X$, $\nu _{x}^\varepsilon \subset V$ where $V'\subset V$ is a possibly smaller neighbourhood of *a*. Now, suppose that for some distinct $x,x'\in V'\cap X$, the segments $\nu _x^\varepsilon$ and $\nu _{x'}^\varepsilon$ do intersect and let *z* be a point in the intersection. The distance *d*(*z*) is realized by a point $x''\in U\cap X$ that may be different from $x,x'$. Since there cannot be $||z-x||=||z-x''||$ because of the univaluedness of *m*, we may assume that $||z-x||>||z-x''||$. But then $x''$ lies inside the biball $b(x,\varepsilon )$ contrary to the definition of $r_a(U)$. Thus, (2) holds.

$(2)\Rightarrow (3)$. Fix $r>0$ such that $\mathbb {B}(a,2r)\subset U$. Then for any $z\in \mathbb {B}(a,r)$, there is $m(z)\subset U\cap X$. If in addition we ask that $d(z)<\varepsilon$, then $\#m(z)=1$, for, given $x,x'\in m(z)$, we necessarily have $z\in \nu _x^\varepsilon \cap \nu _{x'}^\varepsilon$. This gives (3) as $M_X$ does not intersect $\mathbb {B}(a,r)\cap \{d(z)<\varepsilon \}$.

$(3)\Rightarrow (1)$. Assume that (1) does not hold, i.e. $r_a(U)=0$ for all neighbourhoods *U* of *a*. Let us introduce^30^

$$\begin{aligned} r_{\pm \nu }(x):=\sup \left\{ r\ge 0\mid \mathbb {B}(x\pm r\nu (x),r)\cap X=\varnothing \right\} . \end{aligned}$$

By the definition, if $0<r_\nu (x)<+\infty$, then $x+ r_\nu (x)\nu (x)$ belongs to $C_X$. In our case, for any *U*, there is a point $x\in U$ such that either $r_{\nu }(x)$, or $r_{-\nu }(x)$ is finite.

As $r_a(U)=0$ for arbitrary small neighbourhoods *U*, there are two possibilities: either there is a sequence $X\ni x_k\rightarrow a$ such that for any *k*, one of the two radii $r_{\pm \nu }(x_k)$ is finite, positive and it is possible to choose them so that they converge to zero—in which case $a\in \overline{C_X}=\overline{M_X}$, or there is a neighbourhood $U\ni a$ and a constant $C>0$ such that *a* belongs to the closure of the set $U_0:=\{x\in U\mid r_{\nu }(x)=0\ {\text {or}}\ r_{-\nu }(x)=0\}$ whose complement in *U* is $U_C:=\{x\in U\mid r_{\pm \nu (x)}\ge C\}$ 31.

Now, if $a\in U_0$, i.e. one of the radii $r_{\pm \nu }(a)$, say $r_\nu (a)$, is zero, then for any $r>0$, the point *a* is not the closest point to $a(r):=a+ r\nu (a)$. Of course, for small *r*, the point *a*(*r*) has at least one closest point $y(r)\in U\cap X$ and these points must converge to *a* when $r\rightarrow 0^+$, for $a(r)\rightarrow a$. Since $a(r)-y(r)$ are non-zero and normal to *X* at *y*(*r*), we conclude that $r_{\nu }(y(r))>0$. Note that $(a(r)-y(r))/||a(r)-y(r)||=\nu (y(r))$, because the points *a*(*r*) are on the side of *X* designed by $\nu (a)$. If the radii $r_\nu (a(r))$ stayed separated from zero by a constant $c>0$, then since the open balls $\mathbb {B}(y(r),c)$ are disjoint with *X* and converge to $\overline{\mathbb {B}}(a,c)$, we would conclude that $\mathbb {B}(a,c)\cap X=\varnothing$, contrary to $r_\nu (a)=0$. Hence, $r_\nu (y(r))\rightarrow 0$, and so *a* is the limit of the points $a(r)\in C_X$, as required.

Next, suppose that $a\in U_C$. We know that there is a sequence $U_0\ni x_k\rightarrow a$ and, to each $x_k$, we may apply the preceding reasoning in order to conclude that $x_k\in \overline{C_X}$. Therefore, $a\in \overline{M_X}$, too. All these results show that (3) does not hold, and thus the proof is completed. $\square$

### Reaching Radius

The previous subsection suggests some possible notions of *reaching radius* that could detect the approaching of $M_X$. We are looking for a notion of radius of curvature that would detect the medial axis (the usual notion—too local—need not do this even in the ${\mathcal {C}}^2$ smooth situation).

A kind of reach radius that immediately sees the approaching of the medial axis would be the following. Let $N'(X):=\bigcup _{x\in X} N'(x)$. Note that the union is disjoint and this set is the projection onto ${\mathbb {R}}^n$ of the graph of $x\mapsto N'(x)$, hence it is definable. For any $a\in X$, we put

$$\begin{aligned} \rho (a):=\sup \left\{ r\ge 0\mid \overline{\mathbb {B}}(a,r)\subset N'(X)\right\} . \end{aligned}$$

Immediately we obtain

#### Proposition 4.20

For any $a\in X$, $\rho (a)=d(a,M_X)$, and so $x\mapsto \rho (x)$ is definable.

#### Proof

By Theorem 2.7, we know that $M_X={\mathbb {R}}^n\setminus N'(X)$. Therefore, for $a\in X$,

$$\begin{aligned} \rho (a)=\sup \left\{ r\ge 0\mid \overline{\mathbb {B}}(a,r)\cap M_X=\varnothing \right\} , \end{aligned}$$

which readily implies that $\rho (a)=d(a,M_X)$. As we know that *X* and $M_X$ are definable, so is the distance to this set. $\square$

#### Corollary 4.21

For any $a\in X$, the following conditions are equivalent:

1. $$\rho (a)=0$$;
2. $$a\in \overline{M_X}$$;
3. $$a\notin {\mathrm {int}}N'(X)$$.

#### Proof

The equivalence of the two first conditions is clear, for the third one we invoke Theorem 2.7 again. Indeed, ${\mathbb {R}}^n\setminus N'(X)=M_X$, but for any $A\subset {\mathbb {R}}^n$, there is ${\mathrm {int}} A={\mathbb {R}}^n\setminus \overline{{\mathbb {R}}^n\setminus A}$, which accounts for the equivalence between conditions (2) and (3), as ${\mathrm {int}} N'(X)={\mathbb {R}}^n\setminus \overline{M_X}$. $\square$

#### Corollary 4.22

For any $a\in X$, $\rho (a)>0$ iff $\delta (x)=d(x,X)^2$ is of class ${\mathcal {C}}^1$ in a neighbourhood of *a*.

#### Proof

By Clarke’s results and our previous remarks, $\delta$ is of class ${\mathcal {C}}^1$ in a neighbourhood of *a* iff $\partial \delta$ is univalued in this neighbourhood. But this is equivalent to saying that $M_X$ stays away from *a* by Theorem 2.23. $\square$

#### Remark 4.23

As observed in [15], $\delta$ may be of class ${\mathcal {C}}^1$ even at points $a\in {{\mathrm {Sng}}}_1 X$, e.g. for $X=(-\infty ,0]$ in ${\mathbb {R}}$, that is the case.

The number $\rho (X):=\inf \{\rho (a)\mid a\in X\}$ corresponds to the *reach* introduced by Federer (see [14]).

A much more general approach is possible, partially inspired by Theorem 4.19.

Consider a closed, definable, nonempty set $X\subset {\mathbb {R}}^n$. For $a\in X$ and the normal cone $N_a(X)$ we put $V_a:=N_a(X)\cap \mathbb {S}^{n-1}$ 32.

#### Definition 4.24

We define the *weak reaching radius*

$$\begin{aligned} r'(a)=\inf _{v\in V_a} r_v(a) \end{aligned}$$

where

$$\begin{aligned} r_v(a)=\sup \left\{ t\ge 0\mid a\in m(a+tv)\right\} \end{aligned}$$

is the *directional reaching radius* (or *v*-*reaching radius*). Next we put

$$\begin{aligned} \tilde{r}(a)=\liminf _{X\setminus \{a\}\ni x\rightarrow a} r'(x) \end{aligned}$$

for the *limiting reaching radius*. Finally, we define the *reaching radius* as

$$\begin{aligned} r(a)={\left\{ \begin{array}{ll} r'(a), &{}a\in {{\mathrm {Reg}}}_2 X,\\ \min \left\{ r'(a),\tilde{r}(a)\right\} , &{}a\in {{\mathrm {Sng}}}_2 X. \end{array}\right. } \end{aligned}$$

#### Remark 4.25

If $a\in {\mathrm {int}} X$, then $N_a(X)=\{0\}$ and so $V_a=\varnothing$ which gives $r'(a)=+\infty$ (as the infimum over the empty set).

Of course, if *X* is a hypersurface, then at $a\in {\mathrm {Reg}}_1X$, we have $V_a=\{\nu (a), -\nu (a)\}$ where $\nu$ is a local unit normal vector field.

The reason why we consider the biggest lower bound of the radii in all possible normal directions at *a* is to take into account the curvature and obtain a possibly finite number, e.g. for $X=\{y=x^2\}\subset {\mathbb {R}}^2$ we have $r'(0)=r_{(0,1)}(0)=1/2<r_{(-1,0)}(0)=+\infty$.

Taking into account the limiting radius is motivated by the fact that for $X=\{y=|x|\}$ we have ${r}'(0)=+\infty$, while $\liminf _{X\setminus \{0\}\ni x\rightarrow 0}r'(x)=0$. On the other hand, if, e.g. $X=((-\infty ,-1]\cup [1,+\infty ))\times \{0\}$. Then $\tilde{r}(-1,0)=+\infty$, while using the directions from the normal cone, we see that $\inf _{v\in V_0} r_v(-1,0)=1$. This explains the minimum in the definition.

#### Proposition 4.26

Under the assumptions above,

1. for any $a\in {\mathrm {Reg}}_2 X$, there is $r(a)>0$;
2. for any $a\in X$ and any $v\in V_a$ such that $0<r_v(a)<+\infty$, there is $a_v:=a+r_v(a)v\in C_X$;
3. for any $a\in X$ and any $v\in V_a$, $a+tv\in N'(a)$ for all $t\in [0,r_v(a))$.
4. for any $a\in X$ such that $r(a)=+\infty$, there is $N_a(X)+a=N'(a)$.

#### Proof

Ad (1). By assumptions, *X* is a ${\mathcal {C}}^2$ submanifold in a neighbourhood of *a* 33. By the Nash Lemma (cf. [9, Lemma 1.1]), the multifunction *m*(*x*) is univalued in a neighbourhood $U\ni a$. Take $r>0$ such that $\mathbb {B}(a,2r)\subset U$. As $x-m(x)$ is normal to *X* at *m*(*x*), we obtain for any $x\in ((T_a X)^\bot +a)\cap \mathbb {B}(a,r)$, $m(x)=a$. This implies (1).

Ad (2). If $a_v\notin C_X$, then we may increase the ball $\mathbb {B}(a_v, r_v(a))\subset {\mathbb {R}}^n\setminus X=\Omega$ without leaving $\Omega$ but of course keeping the tangency point *a*. This, however, increases $r_v(a)$, contrary to the definition.

Ad (3). This follows directly from our previous considerations.

Ad (4). It is a consequence of the preceding point. $\square$

At this stage, let us consider $X_\infty :=\{a\in X\mid r(a)=+\infty \}$ and $X_+:=X_\infty \cap {\mathrm {Reg}}_1 X$.

#### Lemma 4.27

For $a\in X_+$, we have $X\subset T_a X+a$.

#### Proof

We have $a\in X_\infty \cap {\mathrm {Reg}}_1 X$. Of course, we may assume that $a\notin {\mathrm {int}} X$. Then in any normal direction $v\in V_a$, there must be $r_v(a)=+\infty$. By (3) in the previous Proposition, this means that for a fixed $v\in V_a$, $a+\mathbb {R}_+v\subset N'(a)$, i.e. $X\subset {\mathbb {R}}^n\setminus \mathbb {B}(a+tv,t)$ for any $t>0$. Therefore,

$$\begin{aligned} X\subset \bigcap _{v\in V_a}\left\{ y\in {\mathbb {R}}^n\mid \langle y-a,a+v\rangle \le 0\right\} =T_a X+a \end{aligned}$$

as required. $$\square $$

#### Theorem 4.28

$X_+$ is either void or a union of some connected components of ${\mathrm {Reg}}_1 X$.

#### Proof

It is enough to show that $X_+$ is closed and open in ${\mathrm {Reg}}_1 X$.

For $a\in X_+$, we may assume that $a\notin {\mathrm {int}} X$. Then we have $r(a)=+\infty$ which means that $r_v(a)=+\infty$ for any $v\in V_a$, whence $X\subset T_a X+a$. The point *a* lies in ${\mathrm {Reg}}_1 X$, so that in a neighbourhood *U* of *a*, the set *X* is the graph of a ${\mathcal {C}}^1$ function over $U\cap T_a X+a$. But then $X\cap U=(T_a X+a)\cap U$ which implies that $r\equiv +\infty$ in $U\cap X$ and so $X_+$ is open in ${\mathrm {Reg}}_1 X$.

Take now a convergent sequence $X_+\ni a_\nu \rightarrow a\in {\mathrm {Reg}}_1 X$. Since we have $X\subset T_{a_\nu }X+a_\nu$ and $T_{a_\nu } X$ converges to $T_a X$, we conclude that $r(a)=+\infty$, and so $X_+$ is closed in ${\mathrm {Reg}}_1 X$. This ends the proof. $\square$

#### Example 4.29

It is rather hard to say something about $X_\infty \cap {\mathrm {Sng}}_1 X$ as we can see from the following three examples:

1. Suppose *X* is described by $y\ge |x|$. Then $X_\infty =X$ (note that ${\mathrm {Sng}}_1X$ is the boundary of *X*).
2. Let *X* be the *x*-axis in ${\mathbb {R}}^2$ without the open segment (0, 1). Then apart from the unique singular points (0, 0) and (1, 0) at which it is finite, the radius *r* is infinite. This shows that $X_\infty$ need not be closed in *X*.
3. Let *X* be the *x*-axis in ${\mathbb {R}}^2$ for $x\ge 0$ together with $y=1/x$ for $x>0$. Then along the hyperbola, the radius goes to infinity when we approach the *y*-axis, while at the origin (the unique singularity), it is finite.

Our main aim now is to prove the definability of the newly introduced radius $r:X\rightarrow [0,+\infty ]$. Let us have a look at a general approach. For a definable set, $X\subset {\mathbb {R}}^n$ the multifunction

$$\begin{aligned} \Theta :X\ni x\mapsto C_x(X)\in \mathscr {P}({\mathbb {R}}^n) \end{aligned}$$

is definable (see, e.g. [12]), for its graph $\Gamma _\Theta$ is described by a first-order formula involving definable sets:

$$\begin{aligned}&(x,y)\in \Gamma _\Theta \ \Leftrightarrow \ x\in X, y\in C_x(X) \\&\quad \Leftrightarrow \ x\in X, y\in \limsup _{t\rightarrow 0^+}\frac{1}{t}(X-x)\\&\quad \Leftrightarrow \ x\in X, \forall r>0,\ \forall \varepsilon >0, \ \exists t\in (0,\varepsilon ),\ \exists z\in \hat{X}_{(x,t)}:||y-z||<r \end{aligned}$$

where $\hat{X}_{(x,t)}$ is the (*x*, *t*)-section of the definable set

$$\begin{aligned} \hat{X}=\left\{ (x,t,z)\mid x\in X, t>0, tz+x\in X\right\} . \end{aligned}$$

Similarly, the multifunction

$$\begin{aligned} \theta :X\ni x\mapsto N_x(X)\cap \mathbb {S}^{n-1}\in \mathscr {P}({\mathbb {R}}^n) \end{aligned}$$

is definable. From this, we obtain the definability of the weak reaching radius

$$\begin{aligned} r'(x)=\inf _{v\in \theta (x)}\sup \left\{ t\ge 0\mid x\in m(x+tv)\right\} , \quad x\in X, \end{aligned}$$

as introduced in Definition 4.24:

#### Proposition 4.30

The set $X'_\infty =\{x\in X\mid r'(x)=+\infty \}$ is definable and so is the function $r'|_{X\setminus X'_\infty }$.

#### Proof

This follows directly from the descriptions and the definability of $\theta$ and *m*. Indeed,

$$\begin{aligned} r'(x)=&+\infty \\&\Leftrightarrow \ \theta (x)=\varnothing \ \vee \ \forall v\in \theta (x),\ \sup \left\{ t\ge 0\mid x\in m(x+tv)\right\} =+\infty \\&\Leftrightarrow \ \theta (x)=\varnothing \ \vee \ \forall v\in \theta (x),\ \forall t\ge 0, x\in m(x+tv), \end{aligned}$$

which accounts for the definability of $X'_\infty$.

Observe that $s\le \sup \{t\ge 0\mid x\in m(x+tv)\}$ is equivalent to saying that $x\in m(x+sv)$. Hence, $r'(x)=r<+\infty$ is equivalent to the condition

$$\begin{aligned} \forall v\in \theta (x),\ x\in m(x+rv) \ \wedge \ \forall \varepsilon >0, \ \exists v_\varepsilon \in \theta (x):x\notin m\left( x+(r+\varepsilon )v_\varepsilon \right) , \end{aligned}$$

which gives the definability of $r'$ on $X\setminus X'_\infty$. $\square$

This settles the definability of $r'(x)$, $x\in X$. Finally, we apply the following general fact:

#### Proposition 4.31

Let $E\subset {\mathbb {R}}^n$ be a closed, definable set and $\varphi :E\rightarrow [0,+\infty ]$ a definable function. For a closed, definable, nonempty $F\subsetneq E$ satisfying $\overline{E\setminus F}=E$, we put

$$\begin{aligned} \psi (y)=\liminf _{E\ni x\rightarrow y, x\ne y}\varphi (x),\quad y\in F. \end{aligned}$$

Then $\psi$ is a definable function.

#### Proof

We observe that for $y\in F$,

$$\begin{aligned} \liminf _{E\ni x\rightarrow y, x\ne y}\varphi (x)=\inf (\overline{\Gamma _{\varphi |_{E\setminus \{y\}}}})_y \end{aligned}$$

where $Z_y$ denotes the *y*-section of the set *Z* and $\Gamma _{\varphi |_{E\setminus \{y\}}}$ is the graph of the restriction $\varphi |_{E\setminus \{y\}}$. Consider the definable set

$$\begin{aligned} G=\left( \Gamma _\varphi \times F\right) \setminus \Delta \end{aligned}$$

where $\Delta =\{(x,t,y)\in E\times [0,+\infty ]\times F\mid x=y\}$. Then for a fixed $y\in F$, the section $G_y=\{(x,t)\mid (x,t,y)\in G\}$ is $\Gamma _{\varphi |_{E\setminus \{y\}}}$.

Next, we observe that the set

$$\begin{aligned} H&=\left\{ (x,t,y)\in E\times [0,+\infty ]\times F\mid (x,t)\in \overline{G_y}\right\} \\&=\left\{ (x,t,y)\mid \forall r>0,\exists (x',t'):(x',t',y)\in G\ \wedge \ \left| \left| (x,t)-(x',t')\right| \right| <r\right\} \end{aligned}$$

is definable. Therefore, the multifunction

$$\begin{aligned} h:Y\ni y\mapsto H_y\in \mathscr {P}(E\times [0,+\infty ]) \end{aligned}$$

is definable, too. Note that the *y*-section $h(y)_y$ is exactly $(\overline{\Gamma _{\varphi |_{E\setminus \{y\}}}})_y$.

Finally, we have $\psi (y)=\inf h(y)_y$ which ends the proof. $\square$

#### Theorem 4.32

In the situation considered, the function $r:X\rightarrow [0,+\infty ]$ is definable.

#### Proof

From Proposition 4.31, we obtain the definability of $\tilde{r}(x)$, $x\in {\mathrm {Sng}}_2X$ which together with Proposition 4.30 yields the definability of $r(x)=\min \{\tilde{r}(x),r'(x)\}$, $x\in {\mathrm {Sng}}_2 X$ and means that *r*(*x*) is definable as a function of $x\in X$. $\square$

We end with some more general properties of the introduced radius.

#### Theorem 4.33

Under the assumptions above,

1. the set $X_\infty$ is definable and $M_X\ne \varnothing$ implies $X\setminus X_\infty \ne \varnothing$;
2. the function $\hat{r}:X\setminus X_\infty \ni a\mapsto r(a)\in [0,+\infty )$ is definable and $\hat{r}^{-1}(0)\subset {\mathrm {Sng}}_2 X$;
3. for any $x\in C_X$ and any $a\in m(x)$, $d(x,X)\ge r(a)$.

#### Proof

Ad (1). The definability of $X_\infty$ is a consequence of the definability of *r*(*x*) proved in the last Theorem. For a point $x_0\in M_X$ and any $y\in m(x_0)$, we have $r'(y)\le r_v(y)=d(x_0)<+\infty$ where $v=(x_0-y)/||x_0-y||$. Thus, $y\in X\setminus X_\infty$.

Ad (2).This is a consequence of the previous considerations and Proposition 4.26 (1).

Ad (3). We have $[a,x)\subset N'(a)\subset N_a(X)+a$ and, obviously, also $r_{\frac{x-a}{||x-a||}}(a)=d(x,X)$ whence the result. $\square$

The next and last theorem is related to Theorem 4.19. We would like to obtain an equivalence between the reaching of the point $a\in X$, i.e. $a\in \overline{M_X}$, and the vanishing of the radius: $r(a)=0$. There are several problems that make this difficult.

#### Remark 4.34

It would be helpful, if $r'(a)=0$ implied $\tilde{r}(a)=0$. Unfortunately, the example of $X=\{(x,y,z)\mid z=0, y\le |x|^{3/2}\}$ shows that there may be $r'(a)=0$ and $\tilde{r}(a)>0$. Here ${\mathrm {Sng}}_1 X=\{(x,|x|^{3/2},0)\mid x\in {\mathbb {R}}\}$, so that $r'\equiv +\infty$ along ${\mathrm {Reg}}_1 X$.

On the other hand, if *X* is the graph of $f(x,y)=y|x|^{3/2}$, then $r'(0)>0$, while $\tilde{r}(0)=0$, in particular, $r'(0,y)=0$ for $y\ne 0$.

#### Theorem 4.35

In the setting considered, $a\in \overline{M_X}$ iff $r(a)=0$.

#### Proof

If $a\in {\mathrm {Reg}}_2 X$, then $a\notin \overline{M_X}$, by the Nash Lemma ([9, Lemma 1.1]), and necessarily $r'(a)>0$. Therefore, we assume for the rest of the proof that $a\in {\mathrm {Sng}}_2 X$.

Consider $X_\infty =\{x\in X\mid r(x)=+\infty \}$ and let $W:=\{x\in {\mathbb {R}}^n\mid m(x)\subset X\setminus X_\infty \}$. Equivalently, *W* is described by the condition $m(x)\cap X_\infty =\varnothing$. Observe that $M_X\subset W$. Moreover, $M_X\ne \varnothing$ (which is the case of interest to us) implies $X\setminus X_\infty \ne \varnothing$.

Take now a point $x\in W\setminus X$ and a point $y\in m(x)$. Then $0\ne x-y\in N_y(X)$ and for $v(x,y):=\frac{x-y}{||x-y||}$ we have $r_{v(x,y)}(y)\in (0,+\infty )$. Therefore, the point

$$\begin{aligned} \eta _y(x):=y +r_{v(x,y)}(y)v(x,y) \end{aligned}$$

is well-defined. It is obvious that $\eta _y(x)\in C_X$. Actually, $x\in M_X$ iff for some (and then for any) $y\in m(x)$, $\eta _y(x)=x$. In this case, $r_{v(x,y)}(y)=d(x)$.

If $a\in \overline{M_X}=\overline{C_X}$, then there is a sequence $(x_\nu )\subset W\setminus X$ such that $\eta _{y_\nu }(x_\nu )=x_\nu$ for some $y_\nu \in m(x_\nu )$ and $x_\nu \rightarrow a$. Then by the semicontinuity of *m*, $y_\nu \rightarrow a$. There are two possibilities: either $y_\nu =a$ for any $\nu$, in which case $r'(a)=0$ and so $r(a)=0$, or (passing to a subsequence) $y_\nu \ne a$, in which case $\tilde{r}(a)=0$ and $r(a)=0$, as required.

Now, let $r(a)=0$. We will show that $a\in \overline{M_X}$. Since $r(a)=\min \{r'(a),\tilde{r}(a)\}$, we have two cases to consider.

(1) $r'(a)=0$. According to the definition $\inf \{r_v(a)\mid v\in V_a\}=0$ and there are two possibilities.

(a) There is a sequence $(v_\nu )\subset V_a$ that we may assume to converge to some $v\in V_a$ (for it lives in $\mathbb {S}^{n-1}$) and such that the directional radii $r_{v_\nu }(a)\rightarrow 0$ but $r_{v_\nu }(a)>0$. Then the corresponding points $a+r_{v_\nu }(a)v_\nu$ belong to $C_X$ and converge to *a*. Hence $a\in \overline{C_X}=\overline{M_X}$.

(b) There is a decomposition $V_a=V_a^0\cup V_a^1$ with $V_a^0\ne \varnothing$ and such that $r_v(a)=0$ for all $v\in V_a^0$, whereas $r_v(a)\ge c>0$ for all $v\in V_a^1$ where $c>0$ is some constant. In this case, we pick a vector $v\in V_a^0$ and observe that $a\notin m(a+tv)$ for all $t>0$. However, we may choose $y(t)\in m(a+tv)$ and we get $y(t)\rightarrow a$ as $t\rightarrow 0^+$. Moreover, it follows also that for $v(t)=(a+tv-y(t))/||a+tv-y(t)||$ we get $r_{v(t)}(y(t))$ positive and finite.

*Claim.* $$v(t)\rightarrow v$$ as $$t\rightarrow 0^+$$.

Suppose the claim holds true. Then two things can happen.

(i) $r_{v(t)}(y(t))\rightarrow 0$ as $t\rightarrow 0^+$. Then $y(t)+r_{v(t)}(y(t))v(t)$ belong to $C_X$ and converge to *a* which ends the proof.

(ii) There is a constant $C>0$ such that $r_{v(t)}(y(t))\ge C$ for all $t>0$. Then the open balls $\mathbb {B}(y(t)+Cv(t),C)$ do not meet *X* and converge to the closure of $\mathbb {B}(a+Cv,C)$. By the convergence, the latter cannot meet *X* either. But this is a contradiction showing case (1)(b)(ii) cannot happen.

Before we turn to case (2) let us prove the Claim. First let us recall that a normal vector $u\in N_y(X)\cap \mathbb {S}^{n-1}$ at $y\in X$ is called *proximal*, if for some $r>0$, *y* is the unique closest point to $y+ru$ in *X*. This is equivalent to saying that for any $x\in X$, $\langle x-y,u\rangle \le (1/2r)||x-y||^2$. Indeed, $m(y+ru)=\{y\}$ is equivalent to $||y+ru-x||>r$ for any $x\in X\setminus \{y\}$. After squaring both sides, this can be rewritten as $(1/2r)||x-y||^2>\langle x-y,u\rangle$.

In our case, we know that the vectors *v*(*t*) are proximal.

Let us take any decreasing sequence $t_\nu \rightarrow 0$. We may assume that the unit vectors $w_\nu :=(y(t_\nu )-a)/||y(t_\nu )-a||$ converge to some *w*. Of course, $w\in C_a(X)$. Let us denote $\beta _\nu :=\angle (y(t_\nu )-a,v)$.

Then we observe that $\cos \beta _\nu \rightarrow 0$. Indeed, $w\in C_a(X)$ implies that $\langle v,w\rangle \le 0$. If there were $\langle v,w\rangle <0$, then we would have $\langle v, w_\nu \rangle <0$, i.e. $\cos \beta _\nu <0$ for all indices large enough. But this means that if we look at the triangle formed by the points $a,y(t_\nu ),a+t_\nu v$, we have

$$\begin{aligned} d(a+t_\nu v)^2=t_\nu ^2+\left| \left| y(t_\nu )-a\right| \right| ^2-2t_\nu \left| \left| y(t_\nu )-a\right| \right| \cos \beta _\nu >t_\nu ^2, \end{aligned}$$

for the term with the minus sign is negative. But this contradicts our assumption $d(a+tv)<t$ for any *t*.

Now, $v(t_\nu )$ being proximal at $y(t_\nu )$ with any $r\in (0,d(a+t_\nu v))$, we can write

$$\begin{aligned} \langle a-y(t_\nu ),v(t_\nu )\rangle \le \frac{1}{2d\left( a+t_\nu v\right) }\left| \left| a-y(t_\nu )\right| \right| ^2 \end{aligned}$$

which is equivalent to

$$\begin{aligned} \langle a-y(t_\nu ),a+t_\nu v-y(t_\nu )\rangle&\le \frac{1}{2}\left| \left| a-y(t_\nu )\right| \right| ^2\\ \left| \left| a-y(t_\nu )\right| \right| ^2+\langle a-y(t_\nu ),v\rangle t_\nu&\le \frac{1}{2}\left| \left| a-y(t_\nu )\right| \right| ^2\\ \left| \left| a-y(t_\nu )\right| \right| ^2&\le 2t_\nu \langle y(t_\nu )-a,v\rangle \\&=2t_\nu \left| \left| y(t_\nu )-a\right| \right| \cos \beta _\nu . \end{aligned}$$

It follows that $||a-y(t_\nu )||/t_\nu \rightarrow 0$.

It remains to show that this implies that $\langle v(t_\nu ),v\rangle$ converges to 1. Note that $\langle v(t_\nu ), v\rangle \le 1$. Since $d(a+t_\nu v)<t_\nu$, we have

$$\begin{aligned} \langle v(t_\nu ),v\rangle&=\frac{1}{d\left( a+t_\nu v\right) }\langle a+t_\nu v-y(t_\nu ), v\rangle \\&=\frac{1}{d\left( a+t_\nu v\right) }\left( \langle a-y(t_\nu \right) , v\rangle +t_\nu )\\&>\frac{1}{t_\nu }\left( \langle a-y(t_\nu ), v\rangle +t_\nu \right) =\frac{\langle a-y(t_\nu ),v\rangle }{t_\nu }+1, \end{aligned}$$

But $|\langle a-y(t_\nu ),v\rangle |\le ||a-y(t_\nu )||$ and so $\langle a-y(t_\nu ),v\rangle /t_\nu \rightarrow 0$ which ends the proof of the Claim.

(2) $\tilde{r}(a)=0$ (and we may assume now that $r'(a)>0$). Here $\tilde{r}(a)=\liminf _{X\setminus \{a\}\ni x\rightarrow a} r'(x)$. Again there are two possibilities.

(a) There are sequences $X\setminus \{a\}\ni x_\nu \rightarrow a$ and $v_\nu \in V_{x_\nu }$ such that $0<r_{v_\nu }(x_\nu )\rightarrow 0$. By the compactness of $V_a$, we may assume that $v_\nu \rightarrow v_0\in V_a$, and so $x_\nu +r_{v_\nu }(x_\nu )v_\nu$ are points from $C_X$ converging to *a* as required.

(b) For any sequence $X\setminus \{a\}\ni x_\nu \rightarrow a$, the sets $V_{x_\nu }$ decompose as in (1)(b) into $V_{x_\nu }^0\cup V_{x_\nu }^1$, and the constants $c_\nu =\inf \{r_v(x_\nu )\mid v\in V_{x_\nu }^1\}$ do not converge to zero. Then, necessarily, $V_{x_\nu }^0$ are not empty, and we have thus $r'(x_\nu )=0$. By (1), this means that $x_\nu \in \overline{M_X}$ which implies $a\in \overline{M_X}$, and hence, the proof is complete. $\square$

## Footnotes

1 i.e. its graph $\Gamma _m:=\{(x,y)\in {\mathbb {R}}^n\times X\mid y\in m(x)\}$ is a definable set.

2 Note that $N(a)=m^{-1}(a)$, whereas $N'(a)=N(a)\setminus M_X$.

3 Where $(\Gamma _m)_x$ denotes the *x*-section of the set $\Gamma _m$.

4 For compact sets, it is the usual Hausdorff distance, and for closed ones, it is the metric giving the Kuratowski convergence.

5 Indeed, for any $\varepsilon >0$ we choose $\delta >0$ in order to have $|d(x+y)-d(x)-\langle \nabla d(x),y\rangle |\le \varepsilon ||y||$ for $||y||<\delta$. Then $|\langle \nabla d(x),y\rangle |\le \varepsilon ||y||+||y||$, *d* being 1-Lipschitz. Finally $\varepsilon \rightarrow 0$.

6 Indeed, $||v(x)||=||\nabla d(x)||=1$ so that $\langle \nabla d(x),v(x)\rangle =1$ implies $\cos \angle (\nabla d(x),v(x))=1$, too, hence $\nabla d(x)$ and *v*(*x*) have the same direction and orientation.

7 Let us note that Corollary 2.1 in [17] is also cited there without a proof but seems to be false which we discuss in [11].

8 Indeed, the point $a_\varepsilon :=\gamma (d(a)+\varepsilon )$ lies in *U*, hence its distance to *X* is realized in precisely one point. But when we know that $d(a_\varepsilon )=d(a)+\varepsilon$ so that $\gamma$ is the segment $[m(a),a_\varepsilon ]$, we see that on the sphere $\mathbb {S}(a_\varepsilon , d(a_\varepsilon ))$ we already have the point *m*(*a*), so there must be $a_\varepsilon \in N'(m(a))$. In particular, $\mathbb {B}(a,d(a))\subsetneq \mathbb {B}(a_\varepsilon ,d(a_\varepsilon ))$, whence $a\notin C_X$.

9 By *half-space* we mean a set of the form $\{x\in {\mathbb {R}}^n\mid \langle x-v,v\rangle \le 0\}$ for some $v\ne 0$.

10 Since *X* is closed, then for some $r'<r$, $\mathbb {B}(x_0.r')\cap X=\varnothing$. Then for some radius $r'<r''<r$ we will have $\overline{\mathbb {B}}(x_0,r'')\cap \bigcup _{j=1}^k X_j=\partial {\mathbb {B}}(x_0,r'')\cap \bigcup _{j=1}^k X_j\ne \varnothing$ so that $d(x_0)=\min _{j=1}^k d_j(x_0)$.

11 Roughly speaking, if *NX* is the normal bundle of *X*, then the focal points of *X* are exactly those points at which the Jacobian of $(a,v)\mapsto a+v$, from *NX* to ${\mathbb {R}}^n$, vanishes, which means that at this point nearby normals intersect. Along a normal line $(T_aX)^\bot +a$, there are at most $n-1$ focal points located at points $a+(1/k_i(a))\nu (a)$ where $k_i(a)$ is the *i*-th principal curvature and $\nu (a)$ is a unit normal after fixing a unit normal vector field $\nu$ on *X* near *a*.

12 Also in this case the upper bound is attained. If $|f_i(x)|\le c_i||x||^{\theta _i}$ for $||x||\ll 1$ where $c_i>0$ and $\theta _i={\mathrm {ord}}_0 f_i$, then $\max |f_i(x)|\le (\max c_i)||x||^{\min \theta _i}$ whence ${\mathrm {ord}}_ 0 f\ge \min \theta _i$. On the other hand, for the Euclidean norm we have $||f(x)||\le |f_i(x)|$ for any *i*, whence ${\mathrm {ord}}_0 f\le \theta _i$.

13 We even have ${\mathrm {ord}}_0 g_{|f|}\ge {\mathrm {ord}}_0 f\cdot {\mathrm {ord}}_0\gamma$ since $|f(\gamma (r))|\le {\mathrm {const.}}||\gamma (r)||^{{\mathrm {ord}}_0 f}$. But $||\gamma (r)||\le {\mathrm {const.}} r^{{\mathrm {ord}}_0\gamma }$, so that $g_{|f|}(r)\le {\mathrm {const.}} r^{ {\mathrm {ord}}_0 f\cdot {\mathrm {ord}}_0 \gamma }$.

14 If the point (*a*, *b*) is sufficiently close to (*x*, *f*(*x*)), then $d((a,b),\Gamma _f\cap U)=d((a,b),\Gamma _f)$. More precisely, if $U=\mathbb {B}((x,f(x)),R)$, then it is sufficient that (*a*, *b*) be taken in $\mathbb {B}((x,f(x)),R/2)$.

15 Note the definability implies that the two branches $\Gamma _i$ can be seen as graphs with constant convexity.

16 Indeed, in a possibly smaller *B*, the two branches lie entirely outside $N_0(X)\setminus \{0\}$ and so does the region $D_1$ between them.

17 i.e. $\overline{C_\varepsilon }\cap X$ consists of two distinct points, one on $\Gamma _1$, the other on $\Gamma _2$.

18 If *X* is defined by $|y|=x^2$, then the branches $y=\pm x^2$ for $x\ge 0$ are consecutive, but not the branches $y=x^2,x\le 0$ and $y=-x^2,x\ge 0$

19 In Example 3.25, the region delimited by the pair of branches $y=\pm x^2$ contributes to $M_X$ at zero, while the region of the pair $y=x^2$ for $x\le 0$ and $x\ge 0$ does not (although the corresponding medial axis is nonempty).

20 Indeed, suppose that there are two such branches $M_1,M_2$ lying in $D(\Gamma _1,\Gamma _2)$ and pick a point *a* between them. Then it has exactly one closest point to *X*, say $b\in \Gamma _1$. But then $[a,b]\cap (M_1\cup M_2)\ne \varnothing$ and if *c* belongs to the intersection (note that $c\ne a,b$), its distance to *X* is necessarily realized in *b* and in some other point $b'$, but then $||a-b'||<||a-c||+||b'-c||=||a-b||=d(a,X)$ which is a contradiction.

21 If $\ell _1,\ell _2$ are the two half-lines tangent to the branches $\Gamma _1,\Gamma _2$, respectively, and so forming up $C_0(X)$, then the branches can be seen as ${\mathcal {C}}^1$ graphs with constant convexity, defined over a segment $[0,\varepsilon v)$ in the bisector $\ell ={\mathbb {R}}_+ v$ of the angle $\angle (\ell _1,\ell _2)$; of course, it may happen that $\ell _1=\ell _2=\ell$.

22 This can be also obtained from the definability of $x\mapsto \#m(x)$ following from [6]. Note also that both points cannot lie on the same branch of *X*.

23 Note that both may occur: $y^3=x^4$ is ${\mathcal {C}}^1$ regular at zero but superquadratic at this point, cf. Example 3.1.

24 This is true when ${\mathbb {R}}^n\setminus V$ does not contain a half-space.

25 meaning its derivative is Lipschitz.

26 The converse is more delicate as the function $f(x)=\sqrt{x}$ over (0, 1) is not ${\mathcal {C}}^{1,1}$, but $L_a(U)<+\infty$ for a given $a\in U=(0,1)\times {\mathbb {R}}$.

27 Remark that this intersection could lie outside *U* as in the example of $X={\mathbb {R}}\times \{-1,1\}$ for which $r_a=1$, but $L_a=0$ at all points. That is why we need the intrinsic boundedness.

28 By the intrinsic boundedness, $m(b_+) \subset U$.

29 It can be infinite as for the graph of $f(x)=|x|^{3/2}$ at $a=0$.

30 Still with the convention that $\mathbb {B}(y,0)=\varnothing$

31 In connection to this, an interesting example is that of the graph of $f(x,y)=y|x|^{3/2}$ for $y\ge 0$ and $f(x,y)=0$ for $y <0$. Here $r_{\pm \nu }(0)>0$ and $r_{\pm \nu }(x,y)\ge {\mathrm {const.}}$ for $y<0$, but $r_{(0,0,1)}(0,y)=0$ for $y>0$.

32 It may be empty when $N_a(X)$ reduces to zero like when $a\in {\mathrm {int}} X$ or $a=0$ for $X:xy=0$.

33 Recall that for a given $r>0$ and any $x\in \mathbb {B}(a,r)$, the distance *d*(*x*, *X*) is realized in $\mathbb {B}(a,2r)$.
